# Recent Trends in Manufacturing of Thermoplastic Sandwich Structures: A Review

**DOI:** 10.3390/ma19102077

**Published:** 2026-05-15

**Authors:** Amal Alliyankal Vijayakumar, Muhammad Zahid, Stefano G. Corvaglia, Francesca Lionetto, Alfonso Maffezzoli

**Affiliations:** 1 Department of Engineering for Innovation, University of Salento, Via per Monteroni, 73100 Lecce, Italy; francesca.lionetto@unisalento.it (F.L.); alfonso.maffezzoli@unisalento.it (A.M.); 2 Quantum Technologies, Optronics & Material Laboratories, Innovation Labs, Leonardo S.p.A., St. Prov. Grottaglie-Monteiasi 83, 74023 Grottaglie, Italy; muhammad.zahid@leonardo.com; 3 Engineering–Technology Research & Innovation, Aerostructure Division, Leonardo S.p.A., St. Prov. Grottaglie-Monteiasi 83, 74023 Grottaglie, Italy; stefano.corvaglia@leonardo.com

**Keywords:** thermoplastic sandwich structure, fusion bonding, adhesive bonding, manufacturing, recycling

## Abstract

**Highlights:**

- Comprehensive review of thermoplastic sandwich manufacturing routes with emphasis on skin/core fusion bonding.
- Fusion bonding enables recyclable and structurally efficient sandwich structures.
- Single-step, multi-step and in situ manufacturing methods are critically analysed.
- Emerging welding-based methods, additive manufacturing, and automated fibre placement enabled fast and facile sandwich manufacturing.
- Advanced processing routes support complex geometries and automated production.

**Abstract:**

Lightweight thermoplastic sandwich structures have a potential in terms of high specific strength, recyclability, repairability, and reduced manufacturing costs and cycle times, thereby widening their applicability in the aviation industry. However, joining thermoplastic skins to the core is considered a critical process in determining the structural integrity of fully recyclable sandwich systems. Despite rapid technological progress, a comprehensive assessment of manufacturing routes capable of achieving reliable skin/core fusion bonding remains lacking. Therefore, this review critically examines manufacturing techniques for thermoplastic-based sandwich panels, with particular emphasis on advanced processes that achieve effective skin/core fusion bonding. Within conventional manufacturing routes, compression moulding and double-belt lamination have the potential for high-volume production and process automation. Skin/core fusion bonding via in situ core formation enhances manufacturing flexibility, particularly for achieving complex designs. Emerging approaches, including additive manufacturing, automated fibre placement, and welding-based methods, are identified as promising fusion-bonding strategies. This offers enhanced manufacturing simplicity and efficiency by minimising interlinked processing stages and eliminating the need for intricate mould patterns. Future advancements are expected to focus on highly integrated and scalable manufacturing routes capable of simultaneously achieving skin consolidation, in situ core formation, and skin/core fusion bonding within a single process. In particular, continuous welding-assisted manufacturing and additive manufacturing-based approaches are highlighted as promising pathways for improving structural integration, recyclability, and production efficiency in next-generation thermoplastic sandwich structures. Overall, this review provides a structured foundation to guide future research directions and support the development of more efficient, scalable, and structurally reliable thermoplastic sandwich manufacturing technologies.

## 1. Introduction

Recent studies have further highlighted the importance of Life Cycle Assessment (LCA) and Life Cycle Inventory (LCI) methodologies for evaluating the environmental impacts, energy consumption, and carbon footprint associated with composite manufacturing and recycling routes [1]. Such sustainability-driven assessments are becoming increasingly important for the development of next-generation lightweight sandwich structures with reduced environmental impact [2,3]. Thus, sustainability has emerged as a crucial driver in modern manufacturing, with particular emphasis on recycling, disassembling solutions, and the responsible management of materials at the end of a product’s life cycle [4,5]. Conventional sandwich structures, predominantly based on thermoset matrix systems, present significant challenges in this context due to limited recyclability, complex end-of-life processing, and relatively labour-intensive manufacturing routes [4]. Thermoplastic sandwich systems, however, have gained increasing attention across multiple industries owing to their superior damage tolerance, recyclability, and compatibility with thermoforming and automated manufacturing processes [4,6]. These characteristics enable reduced production time, lower environmental impact, and improved cost efficiency compared to thermoset-based counterparts. A typical thermoplastic sandwich structure is composed of three layers: a thick, lightweight core material, two thin, stiff outer skins, and the bonding regions between them, which form a critical interface layer [7,8]. In this configuration, the outer skins are primarily responsible for bearing in-plane tensile and compressive loads. In contrast, the core transmits shear between the skins via the interface, thereby ensuring structural integrity and load distribution.

Despite these advantages, sandwich structures are susceptible to several failure modes, including global buckling, core shear failure, shear crimping, skin wrinkling, core indentation, and skin or core fracture [9]. Among these, skin/core interface debonding is widely recognised as the most critical failure mechanism, as it directly compromises load transfer between structural constituents [10,11,12]. Such debonding may originate from insufficient interfacial bonding during manufacturing or develop under cyclic loading, impact events, or environmental exposure during service [12,13,14]. Consequently, the development of reliable and efficient techniques for joining thermoplastic skins to the core is a central challenge in manufacturing structurally robust, fully recyclable sandwich systems.

Compared with comprehensive reviews of thermoset-based sandwich structures, reviews dedicated to thermoplastic sandwich manufacturing approaches remain relatively limited, despite increasing interest in recyclable and weldable thermoplastic systems. Although significant progress has been made in the development of thermoplastic sandwich structures, the manufacturing strategies used to achieve reliable skin/core bonding remain diverse and fragmented across the literature. Existing studies often focus on individual sandwich processing techniques or specific material systems, without providing a consolidated comparison of various bonding mechanisms and processing parameters. Earlier reviews addressed selected methods of thermoplastic sandwich manufacturing [4,6]; however, recent advancements in fusion-based manufacturing technologies, including modified pressure-induced in situ foaming, welding-assisted bonding, automated fibre placement, and additive manufacturing, have introduced new opportunities for integrated skin/core consolidation. These developments have not yet been comprehensively evaluated within a unified framework. Consequently, there remains a clear need for an updated and systematic assessment of both conventional and emerging manufacturing routes, particularly those capable of achieving effective fusion bonding at the skin/core interface. Therefore, this review critically examines and compares existing and emerging manufacturing approaches for full-thermoplastic sandwich panels, with particular emphasis on interfacial bonding mechanisms, processing efficiency, scalability, and structural performance. By consolidating current knowledge and identifying existing limitations, this work aims to support future research and facilitate the development of scalable and recyclable thermoplastic sandwich technologies.

## 2. Skin/Core Bonding Methods

**Figure 1:**
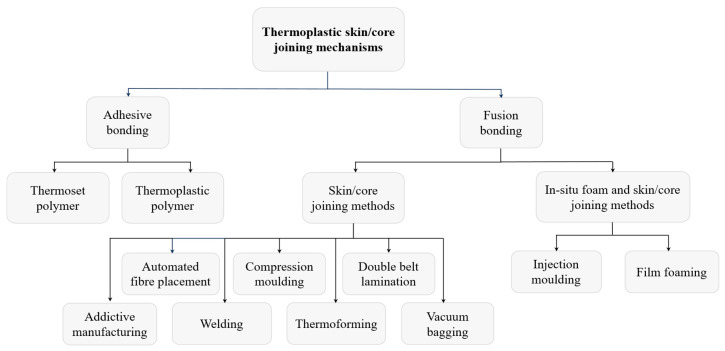
Processing methods used in thermoplastic sandwich structures.

Thermoplastic sandwich panel manufacturing primarily involves bonding the skin and core together. Therefore, skin/core bonding is considered a crucial and critical process in the sandwich construction, determining the structural integrity, load transfer efficiency, and overall performance of the resulting sandwich structure [15,16]. The strength and durability of this interface significantly affect resistance to peel stresses and impact loads, both crucial for static and dynamic applications. Thermoplastic skins to the same polymer core are primarily joined through two bonding methods: adhesive bonding and fusion bonding (also known as fusion welding) [17]. The bonding methods and the corresponding manufacturing adopted in fusion bonding are illustrated in the flowchart shown in Figure 1. Moreover, Table 1 tabulates various single-polymer skin/core sandwich systems with the adopted bonding methods.

Thermoplastic sandwich systems with commodity and engineering polymers, PP, PET, PLA, PC, and PA, were the most commonly explored for the single thermoplastic polymer sandwich systems, according to Table 1. The gained popularity of these polymers is attributed to their low material cost and excellent processability characteristics [4,18,19]. These materials also provide a favourable balance of mechanical performance, such as stiffness and strength, while maintaining relatively low density, making them suitable for lightweight structural applications in automotive, marine, and consumer goods sectors. In contrast, high-performance polymers, such as PEEK, PEI, PPS, and PESU, which are of interest in aerospace and aviation, are not often utilised as mono-polymer thermoplastic sandwich systems due to their high cost and the requirement of elevated processing temperature, which necessitate specialised equipment and increased complexity in manufacturing. Furthermore, processing high-performance polymers can lead to challenges such as warpage, residual stresses, and difficulties in handling materials at higher processing windows. Nonetheless, when cost and processing constraints are secondary to performance requirements, these high-performance polymers provide unmatched structural and environmental durability, making them attractive for demanding aerospace, defence, and high-end engineering applications. Thus, the choice of polymer types directly governs the balance between weight, cost, and performance, allowing thermoplastic sandwich structures to be tailored for specific industrial needs.

Mechanisms in various thermoplastic skin/core adhesive and fusion bonding processes are discussed, with the help of some commercial materials and methods from the literature, in the following subsections.

**Table 1:** Overview of various skin materials, core configurations, and manufacturing methods employed in mono-polymer thermoplastic sandwich systems. Various polymer systems studied are polypropylene (PP), polyethylene (PE), poly-lactic acid (PLA), polyethylene terephthalate (PET), polyamide (PA), polycarbonate (PC), polyetherimide (PEI), polyether ether ketone (PEEK), polyphenylene sulphide (PPS), and polyethersulfone (PESU).

Polymer Matrix	Skin Reinforcements	Core Types	Skin/Core Joining Methods	Bonding Mechanism	References
PP	Natural fibre	Foam or honeycomb	Compression moulding (CM)	Fusion	[20,21]
PP	Glass fibre	Foam	CM	Fusion	[7,12,14,18,22,23,24]
PP	Glass fibre	Honeycomb	CM	Fusion	[24,25,26,27]
PP	Glass fibre	Honeycomb	Thermoforming	Fusion	[28,29]
PP	Glass fibre	Corrugated	Hot-melting	Fusion	[30]
PP	Glass fibre	Foam	Batch foaming	Fusion	[31,32]
PP	Glass fibre	Foam	Injection moulding (IM)	Fusion	[29,33,34,35,36,37]
PP	Glass fibre	Honeycomb	Double-belt lamination	Fusion	[38,39,40]
PP	Glass fibre	Foam or honeycomb	Vacuum bagging (Hot-melt adhesive)	Adhesive	[41,42,43]
PP	Glass fibre	Foam	Batch foaming	Fusion	[13]
PP	Glass fibre	Foam	Continuous USW	Fusion	[11,44]
PE	Glass or natural fibre	Honeycomb	Static ultrasonic welding	Fusion	[45]
PE	PE	Foam	Additive manufacturing (AM)	Fusion	[46]
PLA	PLA	Foam/honeycomb	AM	Fusion	[1,47,48]
PLA	Carbon fibre	Honeycomb	AM	Fusion	[49]
PLA	Carbon fibre	Honeycomb	AM	Fusion	[50,51]
PLA	Carbon fibre	Corrugated	AM	Mechanical interlocking	[52]
PLA	PLA	Honeycomb	AM	Fusion	[53,54]
PLA	Carbon fibre	Corrugated/trapezoidal	Hand layup	Adhesive	[55]
PET	Glass fibre	Foam	CM	Fusion	[56,57]
PET	PET fibre	Foam	CM	Fusion	[58,59,60]
PET	Glass fibre	Honeycomb	AM	Fusion	[61]
PA	Carbon fibre	Foam	IM	Fusion	[62,63]
PA	Carbon fibre	Honeycomb	AM	Fusion	[64]
PA	Glass fibre	Honeycomb	CM	Fusion	[65]
PC	Glass fibre	Honeycomb	Thermoforming	Fusion	[66]
PEI	Carbon fibre	Honeycomb	AM	Fusion	[67]
PEI	Glass fibre	Foam	Thermoforming	Fusion	[68,69]
PEI	Glass fibre	Foam	Batch foaming	Fusion	[4,70,71,72,73]
PEEK	Carbon fibre	Truss or lattice	Thermoforming	Fusion	[74,75]
PEEK	Carbon fibre	Honeycomb	Hand layup	Adhesive	[76,77]
PPS	Glass fibre	Foam	IM	Fusion	[78]
PESU	Glass fibre	Foam	CM	Fusion	[79]

### 2.1. Adhesive Bonding

Adhesive joining represents one of the most established and straightforward techniques for assembling sandwich structures and is increasingly being considered in thermoplastic sandwich construction [80,81]. Adhesive bonding offers an effective alternative when the fusion welding of thermoplastic sandwich structures is not feasible, due to material incompatibility, core sensitivity to processing temperatures, or geometrical complexities. In such cases, adhesive bonding involving the aid of a supplementary thin material layer, especially with a thermoset or thermoplastic-based adhesive film, is used at the interface for joining thermoplastic skins to the core.

**Figure 2:**
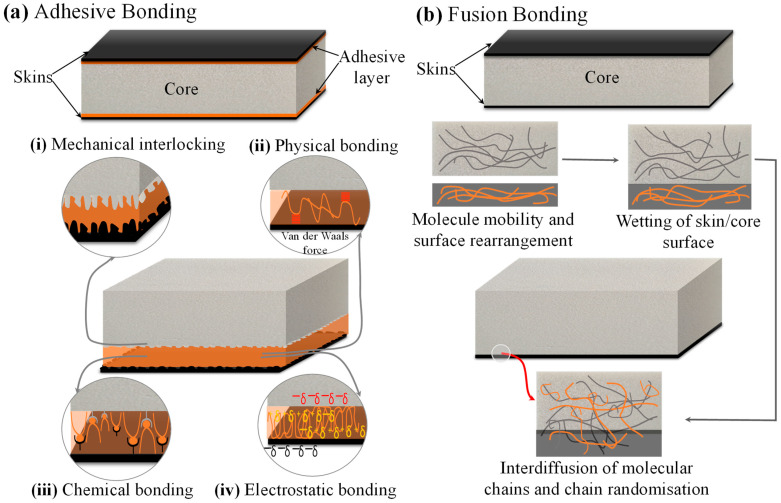
Mechanisms involved in (**a**) adhesive bonding and (**b**) fusion bonding approaches.

Thermoset-based adhesive films are typically epoxy or modified epoxy formulations that cure at moderate temperatures to form rigid and durable joints between thermoplastic matrices [82]. However, thermoset adhesives do not chemically crosslink with the thermoplastic matrix; therefore, mechanical interlocking, chemical bonding, physical bonding and surface activation (via plasma treatment or grit blasting) enable satisfactory adhesion [82,83]. A graphical representation of the mechanisms involved in the adhesive and fusion bonding between thermoplastic skins and core is depicted in Figure 2. Thermoset adhesive use is common in thermosetting-based sandwich structures, providing valuable insights that can be extended to the fabrication of hybrid thermoplastic sandwich configurations. Thermoplastic sandwich skins to the core are typically bonded using thermoset adhesive films through manufacturing methods such as compression moulding, autoclave curing, and vacuum-bag oven curing, all of which apply controlled heat and pressure to soften the adhesive, promote surface wetting and mechanical interlocking with the thermoplastic substrates, and ensure better adhesion of the thermoset resin.

As indicated in Table 1, Rozant et al. [68,69] used 150 g/m^2^ epoxy film, supplied under the commercial name Structufilm R-382 H, for joining single thermoplastic polymer glass fibre-reinforced PEI skins with a PEI foam core. Epoxy film adhesive bonding was used before thermoforming of PEI sandwich structures. The optimal processing window was identified as 165–185 °C for the PEI core and above 280 °C for the skins, with a suitable forming pressure range of 0.03–0.11 MPa. To achieve the required thermal gradient between the skins and core, a two-step heating strategy combined with thermal simulations was developed. Using this approach, the PEI sandwich structure was successfully thermoformed into a hemispherical–ellipsoidal geometry within 7 min. A J-272C adhesive film was used by Hu et al. [74] to bond the CF/PEEK truss core to the CF/PEEK skins. The lap shear joining strength was found to be weak for adhesive bonding (7.35 MPa) compared to the hot-press fusion bonding (17.66 MPa) approach. Likely, the 3D-printed corrugated triangular and trapezoidal core was adhesively bonded to 3D-printed carbon fibre-reinforced PLA skins. The core and skins were printed separately to eliminate the supporting structures formed between the core and skins during the manufacturing process. Consequently, skins were bonded to the core using a two-part epoxy resin (Araldite A/B) [55]. It was noted that cores bonded with skin exhibited a higher compression strength of 1.6 MPa when compared to the core (0.8 MPa). Fareed et al. [84] fabricated a sandwich panel using a 3D-printed poly-lactic acid (PLA) core with different geometries (S-90, S-45, S-V) and self-reinforced polyethene terephthalate (srPET) skins. Before the epoxy adhesion, srPET skins were degreased with ethanol and abraded with silicon carbide sandpaper to slightly rough up the surface, which enhances the wettability and adhesion. Among the investigated core geometries, the S-90 sandwich configuration exhibited the highest fracture strength (52.4 MPa) and tangent modulus (2860 MPa), while the S-V structure showed the highest fracture strain. The results demonstrated improved load transfer and promising mechanical performance for lightweight structural applications. Similarly, Bragagnolo et al. [85] reported the use of a thick epoxy resin layer as an adhesive to join composite skins to a closed-cell polymeric foam core, which delayed the initiation of debonding at the interface. Several hybrid sandwich configurations, involving a thermoset skin with a thermoplastic core, and vice versa, have also been reported, utilising thermoset adhesive bonding [86,87,88,89]. Overall, adhesive bonding remains a versatile joining approach for thermoplastic sandwich structures, particularly for complex geometries and additively manufactured components where direct fusion bonding may be difficult to implement. Epoxy-based adhesive films and resins were the most commonly employed joining systems due to their good processability and compatibility with different thermoplastic composites. However, limitations exist, including the inability to repair debonding, difficulty in recycling at the end of life, a long curing cycle, and extensive surface preparation [4,90]. Furthermore, the potential dissimilarities in thermal expansion and surface energies between thermoset adhesive and thermoplastic substrate materials make the skin/core bonding relatively weak [89,91,92]. Nevertheless, thermoset-based adhesive joining remains a practical and reliable route for manufacturing thermoplastic sandwich structures where other bonding methods are impractical.

An alternative method for joining the skins to the core in sandwich structures is the use of thermoplastic hot-melt films. Thermoplastic hot-melt films are commonly classified under adhesive bonding due to the presence of a distinct interlayer. However, their bonding mechanism may also involve partial polymer chain interdiffusion depending on processing temperature, pressure, and material compatibility. Therefore, they can be considered a transitional or hybrid case between adhesive and fusion bonding. In this method, a thermoplastic polymer film is placed between the skin and the core of the same or dissimilar polymers, softened by heat, and then solidified upon cooling and applying pressure, forming an adhesive layer that bonds the two components. Generally, thermoplastic adhesives have a lower processing temperature than the skin-core polymers. This enables the substrates to remain in their solid state during the bonding process, so that no molecular interdiffusion takes place at the interface. Compared to conventional thermoset adhesives, thermoplastic films offer several advantages: they provide better chemical and thermal compatibility within the thermoplastic skins and core, reducing interfacial stresses caused by differences in surface energy and thermal expansion. Furthermore, the bond is modifiable and reversible, as reheating allows the film to remelt without any chemical degradation. The method is also faster and environmentally friendly, making it a remarkable alternative to the thermoset adhesive technique. Thermoplastic hot-melt adhesive films are processed through manufacturing methods, such as compression moulding, vacuum-bag, or microwave/induction activation.

Wang et al. [93] proposed for the first time the preparation of a full thermoplastic glass fibre/PET foam sandwich panel using a vacuum infusion process with a mixed thermoplastic liquid resin of polymethyl methacrylate (PMMA) and methyl methacrylate (MMA). After the infusion of PMMA to the whole skin/core stack, a two-stage curing process was employed, involving an initial cure at 30 °C for 8 h followed by post-curing at 80 °C for 2 h. Compared with thermoset epoxy systems processed under optimal conditions, the PMMA-based thermoplastic sandwich panels exhibited improvements of 7.64% in flatwise compressive strength and 7.83% in edgewise compressive strength with an adhesive interface failure. Gao et al. [43] developed a 100% recyclable thermoplastic sandwich structure using continuous glass fibre-reinforced polypropylene (GF/PP) skins and a PP circular honeycomb core, assembled using thermoplastic hot-melt adhesive films with 160 μm thickness. The adhesive film was a fibrous network with a melting temperature of 140 °C. Similarly, Cabrera et al. [41] developed a fully recyclable all-PP sandwich panel using a low-melting PP copolymer film as the adhesive layer. The film enabled bonding of self-reinforced PP and glass-fibre-reinforced PP skins to PP foam and honeycomb cores at 135–145 °C and 0.04 MPa, without altering the core or skin structure. Panels were produced using either a press or a vacuum setup. Peel tests showed that adding the hot-melt film significantly improved skin-to-core adhesion, achieving average peel strengths of about 8 N/cm for both foam and honeycomb configurations. Ning et al. [42] designed a body panel using an E-glass fibre/PP skins and PP honeycomb core sandwich composite with hot-melt film adhesive bonding. The adhesive failure leads to the debonding of the exterior and interior skins of the body panel segment. Lee and Ji et al. [94] utilised a thermoplastic polyolefin adhesive film to adhere two different thermoplastic polymer materials, like glass fibre-reinforced polyamide 6 (PA6) skins and an AIREX^®^ T10 (Airex AG, Sins, Switzerland), polyethene terephthalate (PET) closed-cell foam core. The thermoplastic-type adhesive film maintained chemical and physical compatibility with the sandwich constituents and thus attained strong skin-to-core bonding.

Sandwich panels made of glass fibre-reinforced polyetheretherketone (PEEK) skins and a 3D-printed polyetherimide (PEI) honeycomb core were introduced by Martin et al. [95]. A susceptor film made of PEI and µm-sized nickel (Ni) particles was used to generate heat by magnetic hysteresis losses during a vacuum-assisted induction welding process. Likely, Pappada et al. [8] adhesively bonded Twintex^®^ (e-glass/PP) skins to a PET foam core using a low-melting PET copolymer adhesive (BEMIS 5250). This multi-thermoplastic sandwich structure was manufactured using a vacuum bagging and compression moulding process by melting the film at a temperature of 160–180 °C. The results demonstrated that panels manufactured using compression moulding have a higher fracture toughness of 100 J/m^2^ compared with the vacuum bagged sandwich panel (30 J/m^2^). In another study, E-glass/PP skins and HDPE foam with closed-cell surfaces were melted using an infra-red heating with a cold press [96]. Two tie-layer films, namely ethylene-propylene copolymer (EPC) and an HDPE/elastomer blend, were used as hot-melt adhesives for bonding the substrates. With the tie-layer-based sandwiches, a mixed mode of failure was observed in the failed lap shear samples: about 40% is cohesive failure through the tie layer, and the rest of the failure was adhesive either at the PP skins or HDPE surfaces. However, despite the benefits, thermoplastic hot melt films have some limitations. The bond strength is often limited by the mechanical wetting and flow of the molten film, which may not achieve the same intrinsic strength as a fully fusion-bonded interface [42]. Under demanding loading conditions, such as high interlaminar shear, peel, bending, impact, or fatigue loading, this may result in reduced skin/core adhesion and an increased risk of interfacial debonding, consequently necessitating the adoption of fusion bonding (welding) techniques to achieve fully integrated, high-strength joints.

### 2.2. Fusion Bonding

An alternative method of skin/core joining is via fusion bonding, also known as fusion welding. This method is the most familiar technique for joining thermoplastic skins to the core of the same or different miscible polymer types, utilising heat and pressure. Table 1 displays different mono-polymer, from commodity to high-performance, skin/core systems using fusion bonding for sandwich construction. This approach is considered the most efficient joining method for sandwich manufacturing, formed by the interdiffusion of molecular chains at the skin/core interface [7,8,90]. This differs from adhesive bonding, in which the adherents are joined via the mechanisms shown in Figure 2a, and the mechanisms involved in the fusion bonding of the skin/core polymers are pictured in Figure 2b.

The autohesion or self-adhesion theory can be referred to to explain the mechanisms involved in the fusion bonding in thermoplastics [97]. The surface of both the thermoplastic adherents (skin and core) is melted; as a result, the molecules near the surface become mobile, and the bonds are developed as a consequence of surface rearrangement, wetting, diffusion, and randomisation of polymers [90,98]. This interdiffusion of the molecular chains results in an interface joint with the bulk properties of the adherents [4]. The thermoplastic adherents may be either identical or different, and the choice of different adherents is typically determined by the miscibility parameter, which governs the thermodynamic compatibility of polymer pairs [99]. Miscibility determines whether molecular chains can interdiffuse across the interface during fusion bonding. Polymers with closely matching solubility or interaction parameters exhibit favourable miscibility, enabling sufficient chain mobility and interpenetration to form interface joints. For example, PEEK/PEI, PEEK/PEEK, PP/PP, and PPS/PPS combinations exhibited strong interfacial bonding with cohesive substrate failure, indicating good miscibility. PEEK/PPSU showed partial miscibility with an average peel force of ~35 N, whereas PEEK/PES, PPS/PEI, PPS/PES, and PPS/PPSU exhibited poor miscibility and weak interfacial adhesion, with peel forces around 2 N [100]. Fusion bonding of dissimilar polymers is challenging due to their generally poor thermodynamic miscibility, mismatched melting temperatures, incompatible melt viscosities, and differing thermal expansion behaviours [4,90,98]. These factors limit better molecular interdiffusion and generate residual stresses at the interface, often resulting in weak or unstable joints unless compatibilisers or tailored processing conditions are used [101,102]. However, in limited non-structural applications, such as for packaging, a combination of different polymers is still in use. Therefore, the fusion bonding between the skins and the core structure of the same thermoplastic type is far more feasible, as supported by the autohesion theory. Autohesion exhibits molecular compatibility and chain mobility, both of which are maximised when the adherends are chemically the same, enabling strong interdiffusion and the formation of high-quality joints.

This method offers several benefits over adhesive bonding or mechanical fastening, including reduced processing complexity, elimination of supplementary materials, improved recyclability, and shorter cycle times, while also providing high bond strengths [90,98]. Fusion bonding is therefore widely adopted in applications such as thermoplastic sandwich panels, composite laminates, piping systems, and high-rate packaging processes, where rapid, reliable, and fully thermoplastic joints are required. Apart from these advantages, some challenges of fusion bonding have been highlighted. The process is highly sensitive to the temperature and pressure conditions. Inadequate heating leads to insufficient melting and poor fusion bonding, while excessive temperatures can collapse the low-density core, degrade the polymer, or produce uncontrolled melt flow, creating thickness variations or flash [103,104]. Thermal residual stresses may develop during cooling because of non-uniform heat distribution or differences in crystallisation kinetics across the interface, potentially leading to warpage or delamination [90]. Therefore, the selection of this complex processing window possessing a critical surface melting temperature of the adherents (skin/core) and a skin/core consolidation pressure necessary to avoid the core collapse must be carefully optimised for better skin/core bonding.

## 3. Sandwich Manufacturing Techniques

The performance of the skin/core fusion bonding relies mainly on the process window adopted in the chosen thermoplastic sandwich manufacturing methods, and this window varies with the technique employed. Therefore, the manufacturing of the thermoplastic sandwich structure is considered the critical and crucial process in determining the structural performance. The chosen manufacturing route governs the thermal and mechanical histories experienced by the skins and the core, directly influencing interfacial melting, molecular interdiffusion, consolidation quality, and residual stress development. The significance of manufacturing methods lies in their ability to provide controlled and repeatable conditions of several factors, including temperature, pressure, heating/cooling rates, and the dwell time at elevated temperatures, which directly influence the quality of skin/core consolidation and final bond strength. Several 2D and 3D mono-polymer sandwich manufacturing methods resulting in fusion bonding, employed by various researchers/manufacturers, were tabulated in Table 1 and Figure 1. Different manufacturing techniques adopted for thermoplastic sandwich structures are:

1. Compression moulding
2. Vacuum bagging
3. Double belt lamination
4. In situ foaming
5. Welding
6. Automated fibre placement
7. Additive manufacturing
8. Thermoforming

These are explained in the subcategories below. Also, Table 2 is added to summarise the advantages and disadvantages of these different sandwich manufacturing techniques.

### 3.1. Compression Moulding

**Figure 3:**
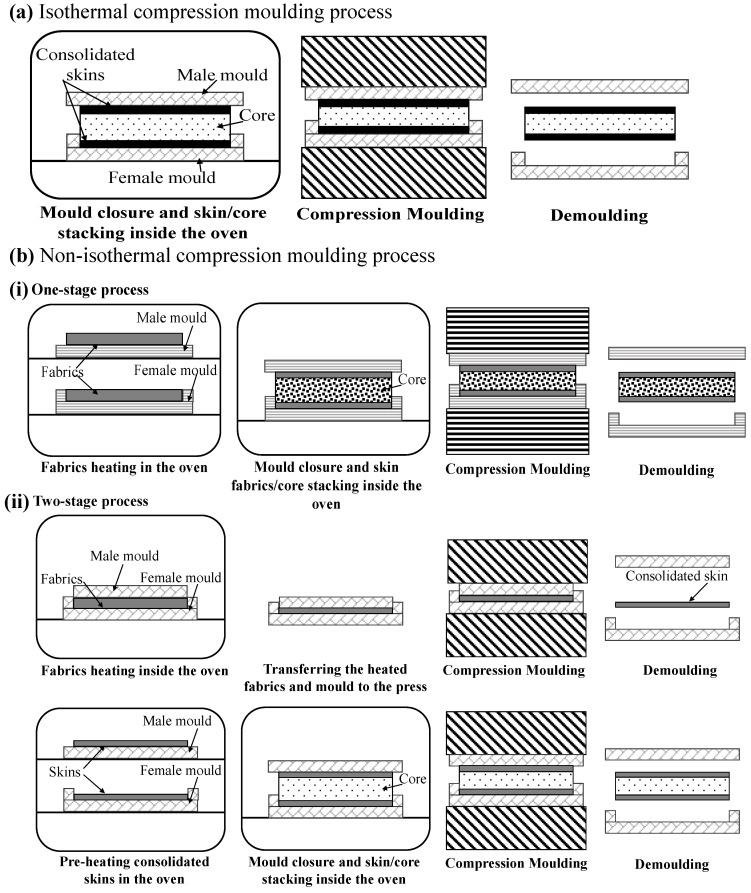
Schematic representations of different compression moulding processes: (**a**) isothermal compression moulding and (**b**) non-isothermal compression moulding.

Thermoplastic sandwich panels manufactured in a mould using a hot press are commonly referred to as the compression moulding process. Several mono-polymer sandwich systems fabricated via compression moulding are illustrated in Table 1. In this process, thermoplastic skins and the core material are stacked in a matched-die mould and subjected to controlled heat and pressure to promote fusion bonding at the skin/core interfaces [4,8]. Lightweight cores with low stiffness are susceptible to the application of high pressure and heat, leading to structural collapse [105]. Compression moulding of sandwich structures is performed mainly in two ways: the isothermal compression moulding process and the non-isothermal compression moulding process. The non-isothermal compression moulding technique is further sub-classified into one-stage and two-stage processes. Different compression moulding methods in manufacturing sandwich structures are schematically shown in Figure 3. Isothermal compression moulding is a process in which the mould, stacked with skin/core, is maintained at a constant temperature, allowing simultaneous skin heating and skin/core consolidation to occur at the interface, as depicted in Figure 3a. In this method, the thermoplastic skins are heated above their melting or softening temperature while pressure is applied, enabling interfacial melting, molecular chain interdiffusion, and consolidation to occur in a single processing step [4]. This approach simplifies the manufacturing cycle and can result in uniform bonding across the sandwich interface.

Isothermal compression moulding presents several demerits when applied to thermoplastic sandwich structures. The process requires the entire mould to be maintained at an elevated temperature, which can lead to long heating and cooling times and consequently increased cycle times and energy consumption. Precise temperature control is critical, as excessive heating may cause polymer degradation, uncontrolled melt flow, or collapse of low-density cores, while insufficient heating can result in incomplete fusion bonding at the skin/core interface. Yellur et al. [25] investigated the isothermal compression moulding of thermoplastic sandwich panels manufactured from glass fibre/PP skins and PP honeycomb cores. The process was carried out at a constant mould temperature of 190 °C, while press force and cycle time were varied. Initial trials using force-controlled pressing, with press forces up to 80 kN and a cycle time of 40 s, resulted in severe honeycomb core damage due to the reduction in compressive strength at elevated temperatures. By employing a position-controlled (zero-force) pressing strategy and an optimised cycle time of 50 s, skin/core fusion bonding was achieved without severe core collapse. The manufactured sandwich panels successfully resisted impact loading up to 100 J. Likely, Skawinski et al. [22] examine the processing of a fully thermoplastic sandwich structure (GF/PP skins and a PP foam core) manufactured by isothermal and non-isothermal compression moulding. The two-stage non-isothermal compression moulding was found unsuitable for manufacturing thermoplastic sandwich panels with high surface quality, despite its relevant advantage in terms of cycle time. Cooling during transfer and surface freezing reduced matrix viscosity, preventing smooth PP flow. In contrast, isothermal compression produced aesthetic parts, with forming pressure influenced by foam thickness, density, and temperature. Overheating caused foam collapse, which lowered the forming pressure and degraded surface quality.

Non-isothermal two-stage compression moulding is a well-known and most widely utilised high-volume industrial method of sandwich manufacturing, particularly due to its short cycle time [7,66]. Skins consolidation and preheating, considered the initial stage, followed by bonding of the skins to the core material under controlled temperature and pressure conditions, characterise the two-step process. This sequential process enables effective skin consolidation, precise control of the skin/core interface, and improved manufacturing efficiency, as schematically illustrated in Figure 3(b(ii)). Grünewald et al. [106,107,108] developed a full thermoplastic sandwich panel with PEI foam core, with and without reinforcing PEI rods. The rods were arranged either orthogonally or diagonally to the carbon fibre/PEEK-PEI skin plane to compete with sandwich structures based on Aramid/phenolic honeycomb cores. During two-stage non-isothermal skin/core joining, heat is transferred from the pre-heated skin onto the foam core surface by heat conduction, thus ensuring the softening of the foam core as well as the protruding ends of the rods. This leads to melting and compressing the rods until the skins additionally come in contact with the foam. By softening and melting the rods’ ends, as well as the foam core surface, a fusion bond was created between the skins, rods, and core. By incorporating rods oriented orthogonally or diagonally to the skin plane and fusion-bonded during processing, the compression and shear properties were significantly enhanced, with improvements of up to 1000% and 72%, respectively. Skawinski et al. [22] introduced a roller conveyor to reduce the transfer time of the mould between the heating press and the cooling press. This ensured fusion bonding of the glass fibre/PP skin/expanded PP foam core via two-stage non-isothermal compression moulding. It was found that the developed panels with a core density of 80 Kg/m^3^ and a thickness of 25 mm showed a higher flexural and flatwise tensile load of 440 N and 3067 N, respectively. Guo et al. [27] introduced a novel non-isothermal moulding process to prevent the core cell collapse in glass fibre/PP skin and PP honeycomb sandwich structure. A steel plate was preheated to the target temperature and rapidly placed on the sandwich panel, enabling heat transfer into the skins and adjacent core and inducing a through-thickness temperature gradient. After a defined heating period, the mould was removed, and a weighted steel plate was applied to provide pressure, allowing the thermoplastic sandwich structure to cool and consolidate at room temperature. This non-isothermal moulding process, in which a high-temperature mould is applied to a low-temperature workpiece, produces fully thermoplastic sandwich structures with strong skin/core interfacial bonding (average peel force of 170 N) and good overall deformation resistance (flexural stiffness > 4 N·m^2^), demonstrating its potential as an effective manufacturing route for thermoplastic sandwich panels. McGarva et al. [109] manufactured a sandwich panel with pre-consolidated glass/polyamide 12 skins and a polymethacrylimide foam core using a non-isothermal two-stage compression-moulding process. Panels were manufactured by heating two pre-consolidated skin laminates in an infrared oven to a temperature exceeding the matrix’s melt temperature (189–279 °C). The heated skins with a 0.1 mm-thick Vestamid L1600 bonding film on the top surface of each skin are placed on either side of the core. The stack is quickly transferred to the press at 23 °C and put into the lower mould half. The cooled mould closes rapidly and applies moulding pressure (0.25–1.75 MPa) to re-consolidate the skin laminates and bond them to the core. The mould then opens, and the panel is removed. The resulting skin/core interfacial Mode I toughness under different moulding pressure and melting temperatures ranges from 0.2 to 0.8 N/mm and 0.15–0.5 N/mm, respectively. Similarly, Åkermo et al. [24,26] detailed the cost model involved in non-isothermal compression moulding of fully recyclable PP sandwich panels with the same monolithic skin composites. The cost model indicates that the use of sandwich manufacturing reduces component cost compared to monolithic composites by improving material efficiency. Thereby, extending the economic feasibility of compression moulding to larger components produced in short to intermediate production series.

In an effort to further reduce production costs in non-isothermal compression moulding by shortening cycle times, Passaro et al. [7] introduced a novel method of skin/core joining, referred to as one-stage non-isothermal compression moulding. One-stage non-isothermal compression moulding process, in which simultaneous skin consolidation and skin/core bonding occur during a single process, as shown in Figure 3(b(i)). The authors observed a 20% increase in skin/core adhesion during the flatwise tensile test using the one-stage method compared to the two-stage method, where preformed skins are preheated and bonded to the core in two separate steps. The improved performance was attributed to a better interdiffusion of molecular chains occurring in the one-stage process between the melted, not yet consolidated commingled E-glass/PP skins and the core surface, i.e., PP foam or honeycomb. In a one-stage process, the potential for polymer degradation in the skin is limited by reducing the skin-preforming cycles, as this process benefits from reduced costs and production time. A similar manufacturing method was utilised by Alliyankal Vijayakumar et al. [12,14] with Twintex^®^ skins and PP foam core to analyse the influence of Mode I interfacial fracture toughness under different press plate temperatures. Mode I tests confirmed that crack propagation occurred entirely within the PP foam core across all specimen geometries and SCB configurations, indicating a fully cohesive failure. Press plate temperature significantly influenced the measured Mode I fracture toughness, with higher press plate temperatures results 19% higher fracture toughness than the lower temperature.

Compression moulding is widely used for the fabrication of flat or moderately contoured thermoplastic sandwich panels due to its ability to provide uniform pressure distribution and good dimensional control [110]. The process allows effective consolidation of the skins onto the core, resulting in high-quality interfacial bonding and relatively low void content when the process window is properly optimised. Furthermore, compression moulding is compatible with a wide range of core materials, including thermoplastic foams and honeycomb structures, making it a versatile manufacturing route for sandwich structures. However, the performance of compression-moulded thermoplastic sandwich structures is highly sensitive to processing parameters such as temperature, pressure, dwell time, and heating and cooling rates [7,23,106]. Excessive pressure or temperature can lead to core collapse, polymer degradation, or excessive melt flow, whereas insufficient heating or pressure may result in incomplete fusion bonding and weak skin/core interfaces. Additionally, non-uniform cooling can induce thermal residual stresses, potentially causing warpage or interfacial delamination [4,110,111]. Despite these challenges, compression moulding remains one of the most established and industrially viable manufacturing techniques for thermoplastic fibre-reinforced composites and sandwich structures on a large-volume basis, owing to its low cost, robustness, repeatability, and compatibility with automated processing. Careful optimisation of the processing window is therefore essential to fully exploit the advantages of this method while mitigating its inherent limitations.

### 3.2. Vacuum Bagging

**Figure 4:**
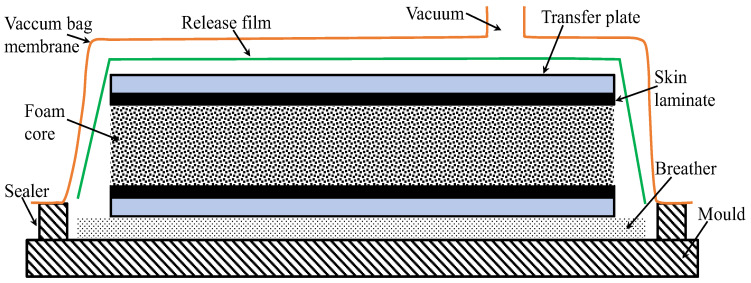
Scheme of vacuum bagging lay-up.

Vacuum bagging is a method of thermoplastic sandwich manufacturing in which the assembled layers of the panel, typically the core and the facing skins, are enclosed within an impermeable membrane or flexible vacuum bag. The schematic arrangement of the vacuum bagging process is pictured in Figure 4. This manufacturing method is derived from the production of thermoset-based sandwich structures [4,110]. Once sealed, air is evacuated from the bag, creating a uniform pressure up to 1 atm over the surface of the panel. This pressure ensures intimate contact between the skins and the core, promoting consolidation and proper bonding while removing trapped air and volatiles. This method is particularly suitable for flat and moderately contoured sandwich panels, where uniform pressure distribution is required [93,110,112]. In addition, vacuum bagging allows for interdiffusion of molecular chains due to the application of heat and pressure in thermoplastic sandwiches, reduced void content, and improved surface finish compared to simple hand lay-up techniques. Releasing films, breather fabrics, and transfer plates are commonly used in the bagging stack to prevent sticking, allow even pressure distribution, and ensure ease of transfer after the heating process [4]. Vacuum bagging is often combined with heat, either via a hot press, heated platen, induction heating or oven, to soften the thermoplastic skins and allow material flow into the core’s surface irregularities, resulting in improved interfacial bonding.

As discussed with the compression moulding, heat conduction across the whole stack in vacuum bagging is achieved via either an isothermal or a non-isothermal approach. The entire stack, including skins, core, auxiliary materials, and tooling, is heated to the desired temperature, along with the simultaneous application of pressure at that temperature, which is known as the isothermal vacuum bagging process [4,8]. Once the target temperature is reached, a dwell period is imposed to allow sufficient softening or melting of the thermoplastic matrix and to promote interdiffusion of molecular chains between the skins and the core. The primary parameters involved in the process are heating temperature, heating/cooling/dwell time, and vacuum pressure. Different mono-polymer sandwich panels manufactured through vacuum bagging are illustrated in Table 1.

The complexity of severe structural core collapse due to the whole stack heating restricted the applicability of isothermal vacuum bagging. Therefore, no literature studies were reported on the sandwich panels made of the same polymer as skin/core components using isothermal vacuum bagging. This process is mainly applied for thermoplastic sandwich manufacturing by means of a hot melt adhesive and discussed in detail in the adhesive bonding section. Pappadà et al. [8] fabricated a full thermoplastic sandwich panel using a high-temperature PET foam as the core and low-temperature Twintex^®^ as skins with and without the aid of low-melting PET copolymer adhesive film through an isothermal vacuum bagging. In this one-stage process, the Twintex^®^ commingled fabrics were directly consolidated and bonded on the PET foam core under vacuum pressure, utilising an oven. Skin/core adhesion primarily occurred due to the PP matrix, which can flow into the foam cells during isothermal processing. The interfacial fracture tests demonstrated that the adhesive film is not capable of enhancing the interfacial fracture toughness when applied under the low pressure exerted by vacuum bagging, due to its limited flow capability in comparison to the low-viscosity PP matrix. Furthermore, the combination of a high-temperature core material with lower-temperature skins effectively limited excessive foam cell collapses during isothermal vacuum bagging.

Heating skins separately from the skin/core bonding under the vacuum pressure is called the non-isothermal process [4,7]. Initially, the thermoplastic skins are first heated separately, for instance, using an oven, typically to a temperature above the glass transition or melting temperature of the polymer matrix, without the application of bonding pressure. Once the desired temperature is reached, the heated skins are then brought into contact with the cold core and transferred to the vacuum table using transfer plates. Then, the membrane is sealed around the stack, a vacuum is created, and the sandwich is consolidated under vacuum pressure. This separation of the heating and bonding stages allows the skins to achieve sufficient matrix softening or melting while limiting heat exposure of the core to the surface, which is particularly advantageous when using temperature-sensitive core materials. As a result, the non-isothermal approach reduces the risk of core deformation, thermal degradation, or cell collapse that may occur when the entire sandwich stack is heated simultaneously. However, the process requires careful control of timing and temperature to avoid premature cooling of the skins before pressure is applied, which could compromise skin/core adhesion. Additionally, the process limits large-scale production attributed to the intensive cost, time, and labour. Grünewald et al. [4] reported non-isothermal vacuum moulding of thermoplastic sandwich panels with glass fibre-reinforced skins and a PP foam core. Uniform skin preheating (180–220 °C) and the use of preheated transfer plates enabled effective consolidation at low vacuum pressures (0.6–0.95 bar). Skin thickness and preheating temperature were identified as the key parameters controlling skin/core adhesion, with an ILSS of 16.5 MPa and a peak three-point bending load of 730 N. Very few attempts were reported on non-isothermal vacuum bagging in a full thermoplastic sandwich manufacturing due to the weak skin/core adhesion, primarily due to the weak skin/core adhesion associated with the low consolidation pressures achievable under vacuum and the temperature losses incurred during material transfer.

### 3.3. Double-Belt Lamination

**Figure 5:**
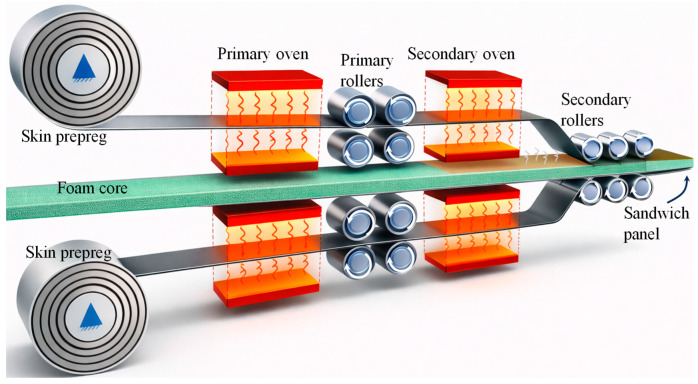
Schematic representation of the double-belt lamination method for thermoplastic sandwich structures (Adapted from [120]).

Double-belt laminating is an automated continuous manufacturing process widely adopted for the industrial production of large thermoplastic sandwich panels based on fusion bonding principles [113,114]. The capacity of uninterrupted operation along the production line makes this approach economically viable. The process employs two sets of primary and secondary rollers in conjunction with the two sets of heating units, commonly referred to as the primary and secondary ovens, as illustrated in Figure 5.

The primary oven is used to heat the thermoplastic composite prepregs to a temperature close to or slightly above the melting or glass transition temperature of the matrix, ensuring uniform softening before consolidation. The preheated skins are thereafter guided through primary rollers, which apply an initial consolidation pressure to stabilise the lay-up and promote preliminary skin laminate consolidation. Subsequently, the assembly of skin/core enters the secondary oven, where additional heat is supplied to fully melt the thermoplastic matrix at the skin/core interfaces. The secondary rollers then apply the main consolidation pressure, enabling effective fusion bonding and thickness control of the sandwich panel before it proceeds to the cooling and solidification stage. Resident time, inlet/outlet clearance, heating and cooling temperatures, roller speed, and applied roller consolidation pressure at the skin/core are the key variables which need careful attention [115,116].

IPCO [117], Kuntai [118], and Lusafe CFRT [119] developed different double-belt lamination models suitable for medium- to large-scale manufacturing of thermoplastic-based foam and honeycomb sandwich panels. The modular configuration of these double-belt press systems allows multiple processing stages, including heating, pressure application, and cooling, to be integrated within a single continuous manufacturing line. Each stage can be independently controlled, enabling precise regulation of temperature and pressure profiles along the processing path. This capability has been demonstrated through the successful manufacture of sandwich panels incorporating highly temperature-sensitive expandable foam cores, even in cases where conventional processing routes were unsuccessful, highlighting the robustness and flexibility of the double-belt laminating approach.

ThermHex and Monopan^®^ lightweight mono-polymer thermoplastic sandwich panels are manufactured with glass fibre/PP skins and PP honeycomb cores using a double-belt lamination process [38,39,40]. A fusion/welding process that homogeneously and permanently joins together the PP skins to the PP honeycomb core was identified. Günter Erhardt [121] describes a PP-based thermoplastic sandwich panel manufactured by double-belt laminating in a patent. In the process, two glass fibre mats are continuously guided through heated impregnation nozzles and pre-impregnated with low-viscosity molten polypropylene, with the melt flows precisely regulated to ensure uniform fibre wetting. Simultaneously, a foamed PP core is produced by extruding polypropylene mixed with a blowing agent and conveyed toward the laminating line. The pre-impregnated mats are stacked on both sides of the foam core and fed into a double-belt press, where the inlet nip is adjustable to pre-calibrate the mats. Within the press, the stack is consolidated under low pressure without adhesives, cooled by opposing cooling plates to reduce residual stresses, and continuously transported to form a fully bonded sandwich panel with controlled density and mechanical properties suitable for vehicle interior applications. ECONCORE NV [122] patented a thermoplastic honeycomb PP sandwich panel manufacturing by double-belt lamination. In the process, unidirectional fibre-reinforced thermoplastic tapes are continuously guided through heated rollers to preheat and soften the material. Simultaneously, a thermoplastic honeycomb core is conveyed along the laminating line. The preheated UD tapes are stacked on both sides of the honeycomb core and fed into a double-belt press, where the sandwich is consolidated under controlled pressure and temperature while continuously cooled to form a fully bonded panel with balanced 0°/90° fibre orientation.

Mandegarian et al. [120,123] fabricated a full thermoplastic glass fibre/PP skin and a 100% recycled PET foam core sandwich panel using the double-belt lamination method. A careful evaluation and comparison of manufacturing parameters with the compression moulding process was carried out. The glass/PP fabric rolls and solid PET core sheets were continuously supplied to the processing line. As the glass/PP fabrics pass through the primary heating chambers, they are heated to approximately 160–165 °C and subsequently consolidated into skin-sheet laminates by a set of rollers. The formed face sheets are then maintained at 160–165 °C, while the PET core is heated separately to 200–205 °C using a secondary oven. In the final stage, the skin sheets are pressed onto the PET core using a secondary set of rollers. The opted processing parameters resulted in the production of high-quality sandwich panels with effective skin/core bonding in the lamination method. The occurrence of combined adhesive and PET core substrate failure during the peel-off and flatwise testing confirmed the development of a strong interfacial bond achieved through the lamination process. A comparison between double-belt lamination and hot-press lamination showed comparable peel and flatwise tensile strengths for the sandwich panels. In double-belt lamination, the peel strength increased from 2810.6 N/m for a 10 mm core to 3364.6 N/m for a 50 mm core, while hot-press lamination exhibited similar values ranging from 2975.5 N/m to 3368.4 N/m. Likewise, the flatwise tensile strength for double-belt lamination increased from 0.76 MPa to 1.08 MPa with increasing core thickness, compared with 0.85 MPa to 1.05 MPa for hot-press lamination. These results indicate that both processes achieved comparable skin/core bonding quality and structural integrity. Although the double-belt lamination technique provides a continuous and fully automated manufacturing route, compression moulding offers significantly more precise control over lamination temperature and pressure.

### 3.4. In Situ Foaming

**Figure 6:**
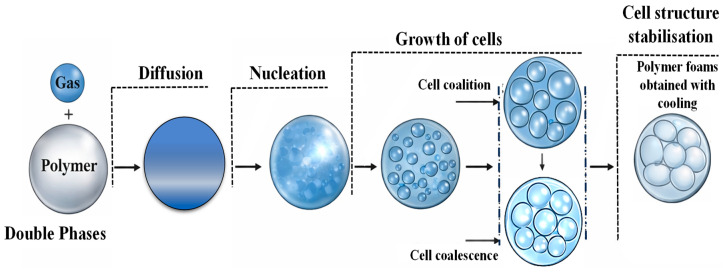
Schematic representation of the microcellular foaming process in polymers, where the lighter shading within the cells indicates a reduced concentration of the blowing agent (Adapted from [131]).

Simultaneous core foaming, followed by the skin/core adhesion, is known as the in situ foaming method. During the in situ foaming, skin/core fusion bonding occurs along with the core formation in the thermoplastic sandwich panels [4,124]. This approach offers several advantages over conventional thermoplastic sandwich manufacturing routes, including the elimination of secondary bonding or adhesive layers, improved skin/core adhesion via melt fusion, fewer processing steps, and enhanced recyclability. In addition, in situ foaming enables precise control of core expansion and panel geometry within the mould, making it suitable for producing components with complex geometries, variable thickness, and integrated functional features [78]. As the name foaming symbolises, the method only allows the production of porous structures from the dense polymer as the core by introducing and expanding a gas phase within the polymer matrix [125]. The polymer foaming method involves several stages, such as gas dissolution, cell nucleation, cell growth, and cell stabilisation and solidification, resulting in a tailored cellular morphology [126]. The process flow for the polymer foaming process is presented in Figure 6. Each stage is critical, as controlling nucleation and growth allows accurate tailoring of cell morphologies, density, and connectivity for different applications ranging from lightweight structural components to thermal insulation. The critical parameters that mainly govern the foaming process are pressure, temperature, foaming time and temperature, and cooling time, as these parameters directly control gas solubility, nucleation, cell growth, and solidification [127,128,129,130].

**Figure 7:**
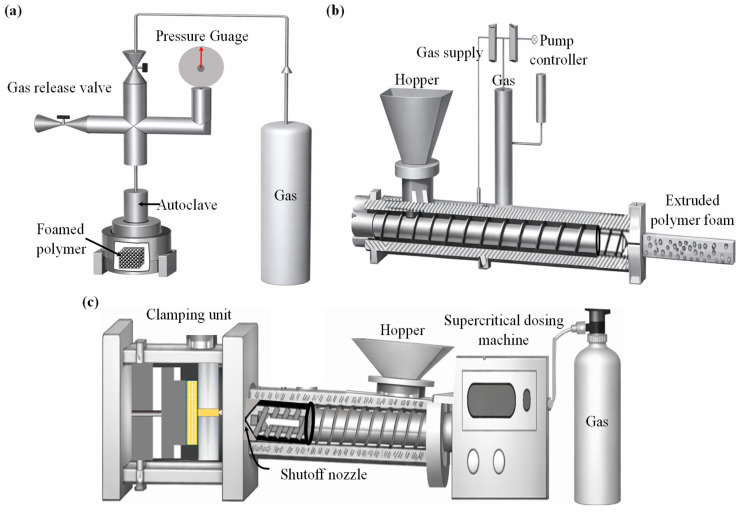
Different foaming approaches: (**a**) batch foaming (pressure-induced), (**b**) extrusion foaming, and (**c**) injection foaming (Adapted from [132]).

The foaming routes specifically used for thermoplastic sandwich core production are batch foaming, extrusion foaming, and injection foaming [132]. The pictorial representations of various predominant foaming routes were depicted in Figure 7. The properties of each foaming route were illustrated in Table 3.

Batch foaming, also known as gas foaming, is a method for generating cell nucleation and cell growth in thermoplastic polymers and thermosets. This method of foaming can be applied in three ways: pressure-induced, temperature-induced, and modified pressure-induced methods [132]. In pressure-induced foaming, the polymer is initially saturated with a physical or chemical blowing agent inside an autoclave under high gas pressure and temperature. Cell nucleation is subsequently initiated rapidly via depressurising the system to atmospheric pressure, referred to as pressure quenching. The subsequent cellular morphology is attained either by quenching the polymer in a cooling medium or by cooling it in ambient air [133]. The pressure-induced batch foaming components mainly include an autoclave for polymer saturation, a pressure/gas releasing valve for pressure quenching, and integrated sensors and gauges for monitoring and controlling pressure and temperature, as shown in Figure 7a. The initial stages for temperature-induced batch foaming, such as the polymer-gas saturation, are similar to the pressure-induced batch foaming, but at a lower temperature and pressure [134]. Later, the gas-saturated polymer is rapidly heated to a temperature above its glass transition or melting temperature while maintaining constant pressure. The increase in temperature reduces gas solubility and polymer viscosity, thereby inducing a supersaturated state that initiates cell nucleation and subsequent growth. Foaming proceeds for a defined foaming time, after which the cellular structure is stabilised by cooling, thereby fixing the final foam morphology.

A modified pressure quenching foaming process has been recently developed, in which the polymer is initially heated to a high temperature under blowing agents to achieve complete melting and gas saturation. The system is then cooled to a predefined foaming temperature at constant pressure, after which cell nucleation and growth are induced by rapid pressure quenching. Although foaming is initiated by depressurisation, the controlled melt-state saturation and cooling steps distinguish this method from conventional pressure-induced batch foaming [130]. Temperature-induced foaming is well-established for thermoplastic sandwich panels, whereas pressure-induced foaming allows better control over expansion and reduces thermal stress. Modified pressure-induced foaming combines thermal activation with controlled pressure release, offering improved foam morphology and mechanical performance, especially for load-bearing structures. The gas-polymer saturation in batch foaming is mainly achieved in a high-pressure autoclave, followed by free or mould-constrained foaming. In hybrid compression-moulding-based batch foaming, gas saturation, nucleation, and growth occur inside a heated mould under controlled closure and pressure quenching, providing precise control over foam geometry, thickness, and cell structure [135].

Brouwer [4] described the fabrication of temperature-induced in situ batch foaming in PEI-based sandwich panels in a two-step process. Initially, precompacted PEI fibre-reinforced skins are combined with a PEI film containing methylene chloride (MC) in a compression-moulded press at elevated temperatures. This creates an initial linkage between the skin laminates and the thermoplastic film. In the subsequent step, the precompact was reheated in a press, causing it to decompact and triggering rapid foaming of the film. The press must be opened to accommodate the expansion of the film, which can increase up to about 16 times its original volume for a PEI film with 17% MC at a foaming temperature of 175 °C. Once the target thickness is achieved, the press is closed, and the sandwich panel is cooled. Finally, the panel is dried to eliminate any residual solvent.

Kluit (1995) [70] proposed a novel in situ film foaming method for the production of PEI-based sandwich panels by featuring an alternative to toxic, carcinogenic blowing agent as MC. In this method, a compression-mould-constrained, temperature-induced batch foaming technique was adopted, in which a physical blowing agent (liquid-based)-impregnated thermoplastic PEI film sheet was foamed between two consolidated prepregs of glass/PEI inside a closed mould under the action of heat and pressure. Once the temperature becomes steady, the press was opened slightly to define the thickness and the density of the foam. This leads to simultaneous core formation, followed by fusion bonding between the skin and core. The sandwich was subsequently cooled to room temperature to make the structure solid and dried inside the oven to remove the residual blowing agent. In this process, PEI film with acetone, as a swelling agent, was placed in a bath, and when the swelling equilibrium has attained, the film was then soaked in the bath with the second liquid. Upon heating, this second liquid, such as ethanol, 1,1,1-trichloroethene, or water, acted as a volatile blowing agent, generating cell nucleation and growth through evaporation. This solvent-assisted in situ foaming route represents a low-cost batch foaming method and can be carried out using liquids that are poor solvents or non-solvents for PEI.

Fits Holding BV (2005) [72] patented a key improvement for Kluit’s method [70] by preventing residual blowing agent escaping from the panel edges during drying, forcing the agent to diffuse through the skins. This ensures homogeneous drying, prevents surface irregularities such as bulges or pits, and also achieves low residual solvent levels (<0.5%), which improves fire resistance and surface quality. Fits Holding BV (2015) [71] patented an advanced compression-mould-based in situ batch foaming technique employing physical blowing agents (acetone), in which a high-rate two-stage cooling strategy is used to overcome the adhesion limitations previously reported for PEI-based sandwich panels by Kluit [70]. The assembly was heated under pressure to a foaming temperature below the core’s glass transition to promote adhesion. Then, the core was expanded by gradually increasing the press spacing, forming a closed-cell, anisotropic foam. The panel was cooled under pressure in two steps: a rapid first cooling (preferably ≥200–240 °C/min) to an intermediate temperature (70–100 °C) to ensure strong adhesion and reduce defects, followed by slower cooling (20–25 °C) to ambient for stabilisation. Finally, the panel was dried at elevated temperatures to remove residual blowing agent. High thermal conductivity layers in the press ensured uniform cooling and consistent panel quality. This method enhanced adhesion and scalability compared to previous methods by Kluit [70], allowing the production of larger, defect-free sandwich panels from a variety of thermoplastic materials.

Martin [73] discloses a patent for a press for in situ manufacturing of thermoplastic sandwich panels with foam cores and fibre-reinforced skins. It explains a fluid circulation loop with a heater and a controlled expansion valve to convert hot, pressurised water into steam, enabling rapid, uniform cooling that prevents foam collapse and ensures a consistent cell structure. The press accommodates both physical and chemical blowing agents and various thermoplastics (PEI, PP, PC, PEEK, PPSU). The method integrates preheating, controlled foaming under pressure, and stepwise cooling in a single cycle, enhancing adhesion, mechanical performance, and reducing cycle times. The novelty lies in the use of expansion-based cooling and precise temperature control, improving homogeneity and foam quality.

FITS Technology, Netherlands [31], reported two low-cost, strong PP-based sandwich panel manufacturing routes using a pressure-induced in situ batch foaming process. The sandwich panels are manufactured via both the in situ foaming and skin/core bonding approach, and the in situ skin consolidation, foaming and skin/core bonding method (known as the low-cost method). The PP film with a chemical blowing agent was stacked between the fibre-reinforced PP fabrics inside a compression moulding press for the low-cost in situ method. These stacks were heated under a higher pressure to consolidate the skins. Subsequently, the entire assembly was cooled to a temperature slightly above the melting temperature of PP. Foaming was then initiated by releasing the pressure and opening the press, allowing activation of the chemical blowing agent and expansion of the core material. A controlled press-opening trajectory was employed to promote the formation of predominantly vertical cellular structures, resulting in sandwich panels with skin/core fusion bonding. Finally, the panel was cooled to stabilise the foamed core structure. Neumeyer et al. [32] investigated processing routes for thermoplastic sandwich structures aimed at automotive serial production, focusing on material-compatible and highly integrated manufacturing concepts. Glass-fibre-reinforced PP skins were used in combination with PP-based foam cores. The skins were attached to the tool surface to fuse the beads between the sandwich surface layers and weld the EPP foam core to the thermoplastic matrix simultaneously, using a bead-foaming approach. The study demonstrates that, under modified steaming conditions, sufficient bead fusion and skin/core adhesion comparable to pressing processes can be achieved. This confirms the suitability of these thermoplastic sandwich concepts for short-cycle automotive manufacturing. Trucillo et al. [13] developed a novel in situ foaming and skin/core-bonding approach via a modified pressure-induced batch foaming process. Temperature and pressure during the process were controlled using a custom-made autoclave to fabricate twintex^®^ skin and PP foam-core sandwich panels. The Mode I characterisation showed no significant effect on foaming for various sandwich panels when 1 °C varied the foaming temperature from 138 °C to 141 °C. The MBT specimens exhibited fracture toughness values ranging from 1874.1 to 2162.8 J/m^2^, while the CDP specimens showed values between 2761.6 and 3205.3 J/m^2^ over the investigated foaming temperature range of 138–141 °C. The relatively large and overlapping error ranges indicate that foaming temperature had no significant influence on the fracture toughness within the investigated processing window.

Batch foaming is primarily employed for research purposes on a laboratory scale, where small sample quantities, typically in the order of a few grams, can be processed. The method is relatively simple to implement and enables precise control over key processing parameters, such as pressure, temperature, and foaming time [134]. Due to its inherently low production rate, only limited industrial applications exist. Nevertheless, the high degree of control over processing conditions and the wide tunability of foaming parameters allow accurate tailoring of the cellular morphology, making batch foaming suitable for high-value-added applications, particularly involving high-performance thermoplastic materials. Furthermore, batch foaming seems beneficial when technical limitations restrict the use of extrusion foaming processing or when specific cellular morphology is desired, such as in the case of biomedical applications. Batch foaming using high-pressure CO_2_ as a physical blowing agent has been highlighted as a more environmentally friendly foaming route, since CO_2_ is non-toxic, chemically inert, and can replace less sustainable chemical blowing agents in polymer foaming processes. Such CO_2_-based foaming has the potential to make large-scale foam production more sustainable by reducing harmful emissions and avoiding chemical residues in the final polymer foam. Batch foaming also permits tuning of foam density across the thickness by manipulating temperature, pressure, and boundary conditions during expansion. Constrained interfaces or controlled temperature gradients result in spatial variations in cell nucleation and growth, leading to graded density and cellular structures, which have been widely reported for solid-state and pressure-induced batch foaming processes [136,137,138].

Injection foaming is also referred to as a semi-continuous process. It is characterised by the foaming of a polymer melt inside a closed mould cavity following injection, where cell nucleation and growth occur under transient pressure and temperature conditions during filling, packing, and cooling [124,125,134]. Injection-moulded polymer foams are most commonly produced using the commercial MuCell^®^ injection-moulding technology, which was developed based on pioneering research conducted at the Massachusetts Institute of Technology [126]. The process enables the production of near-net-shape components with reduced density. However, the short residence time and strong flow-induced effects often limit control over cell uniformity and through-thickness density gradients compared to batch foaming routes. Foam injection moulding follows the same basic principles as conventional injection moulding [33,36,139]. When a chemical blowing agent is employed, it can be introduced directly with the polymer pellets through the hopper during processing. However, when physical foaming is employed, the injection moulding system is equipped with an additional gas delivery unit for introducing the blowing agent (Figure 7c). The gas-filled polymer melt is propelled by the rotating screw and then injected into a sealed mould where foaming occurs, as illustrated schematically in Figure 7c. At present, three main foam injection-moulding technologies are commonly used to manufacture microcellular polymer foams using CO_2_ as a physical blowing agent. These technologies include MuCell^®^ (now commercially known as the MuCell^®^ process), developed by Trexel Inc. (Wilmington, MA, USA), Optifoam^®^, introduced by Sulzer Chemtech AG (Allschwil, Switzerland), and ErgoCell^®^, developed by Demag (Wetter, Germany). These processes represent low-pressure foam injection moulding, in which the mould cavity is partially filled (55–95%) with a short shot of gas-loaded polymer melt [124]. Foam cells nucleate due to the pressure drop at the gate during mould filling, and foaming occurs simultaneously with filling. The resulting foams typically show modest density reductions, often in the range of 10–30% by weight, and exhibit a wide distribution of cell sizes [140]. This behaviour is mainly attributed to the high melt temperature during foaming, the simultaneous occurrence of cell nucleation and melt filling under strong shear conditions, and the restricted time and space available for cell growth within the mould cavity. The cavity pressures in these low-pressure processes generally remain relatively low (0.4–7 MPa). Lohr et al. [141] examined two types of foam injection moulding (FIM) process using glass fibre-reinforced PP, comparing the MuCell^®^ process and a Direct long fibre reinforced thermoplastics (LFT)-foam injection moulding (D-LFT-FIM) process. The D-LFT-FIM process achieved weight-average fibre lengths up to three times longer than MuCell^®^, resulting in improved mechanical properties, particularly impact strength. To address the limitations of conventional foam injection moulding, a novel high-pressure core-back foam injection moulding process has been developed by several research groups [125,126]. The core-back process offers improved foam uniformity, lower density, and better control over cell structure compared to conventional low-pressure foam injection moulding [142,143].

**Figure 8:**
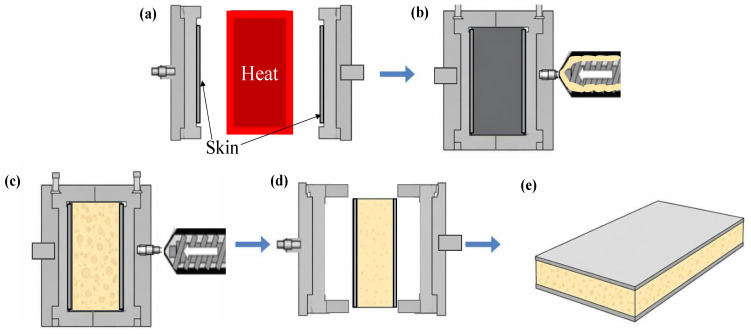
Steps involved in the in-situ injection foaming process of sandwich panels: (**a**) positioning and heating of the consolidated laminate skins inside the mould cavity; (**b**) injection of the gas-loaded PP melt into the closed mould cavity; (**c**) initiation of core foaming through controlled mould expansion and pressure drop; (**d**) final demoulded thermoplastic sandwich structure after cooling; (**e**) in-situ injection foamed sandwich panel.

©OSV polymers [144] has lines for producing polyurethane (PU) foam sandwich panels using OSV L and H series injection machines. Alexander et al. [35] investigated the manufacture of injection-moulded polypropylene-based composite sandwich structures. The skins consisted of unidirectional glass-fibre/PP tapes that were automatically positioned and consolidated using either vacuum consolidation or compression moulding. In the subsequent step, the two consolidated laminates serving as skins were positioned and fixed inside the mould cavity (Figure 8a). To enable fusion bonding with the core material, the surfaces of the skins were indirectly heated (Figure 8a). The mould was then closed rapidly to minimise heat loss, after which a gas-loaded PP melt was injected into the cavity (Figure 8b). Once cavity filling was completed, foaming of the core was initiated by inducing a pressure drop through controlled mould expansion (Figure 8c). The final sandwich thickness was defined by the extent of mould opening. After cooling, the sandwich component was demoulded (Figure 8d). Using this process, sandwich panels with an overall thickness of 6.4 mm and skin thicknesses of 0.26 mm and 1 mm were successfully produced.

In addition, Menrath et al. [36] introduced an alternative processing route combining insert-moulding with foam injection moulding, enabling one-step production of sandwich panels. Prepared skins are positioned in the mould and preheated using quartz infrared emitters on a linear robot. The gas/melt injection is combined with mould embossing to enhance skin/core adhesion. Mould opening controls foaming, core expansion, and final component dimensions. Sandwich panels with 5–6.4 mm thickness were produced using drawn PP fabrics (Curv^®^), PP/glass fibre (glass: 70% wt.) tapes, and unidirectional consolidated sheets, with PP forming the foamed core. The combination of Curv^®^ skins and PP core yields fully recyclable self-reinforced polymer composites. For 6.4 mm-thick sandwich panels with 1 mm skins, density-specific stiffness increased by 130% with self-reinforced thermoplastic skins and by 1900% with glass fibre-reinforced skins. Bending tests and microstructural analysis confirm the process potential, with particular emphasis on preheating and foam morphology.

A thermoplastic melt, such as polypropylene, reinforced with long glass fibres and loaded with a blowing agent (e.g., nitrogen, with 99.99% purity), is injected into the mould cavity. During processing, the material near the mould walls becomes more highly compacted, while the core region develops a lower-density cellular structure. This results in an in situ foamed integral foam exhibiting a pronounced density gradient, giving rise to mechanical characteristics comparable to those of a thermoplastic composite sandwich structure [37]. Likewise, Loypetch et al. [33] fabricated injection-moulded sandwich structures consisting of short glass fibre (30- and 50 wt.%)-reinforced PP skin film layers and a chemically blown PP foam core. The thickness of the film skins, produced by the extrusion process, was 0.39 and 0.55 mm, respectively. The effect of glass fibre content in the skins and blowing agent content in the core was studied with respect to density, microstructure, surface quality, and bending properties. Enhanced skin/core adhesion was achieved during injection moulding. The sandwich structures exhibited reduced foam cell intensity, improved stiffness, and high strength-to-weight ratios due to the stiff glass fibre-reinforced skins and low-density core. Liu et al. [145] investigated the interfacial performance optimisation of continuous glass fibre-reinforced PP sandwich structures manufactured through microcellular injection overmoulding, in which a foamed PP core was directly bonded to continuous glass fibre-reinforced PP skins. The effects of melt temperature, preheating temperature, injection velocity, and foaming agent content on the interfacial fracture behaviour were evaluated using double cantilever beam (DCB) and end-notched flexure (ENF) tests. The results demonstrated that melt temperature and preheating temperature were the most influential processing parameters affecting the Mode I and Mode II interfacial fracture energies. Optimal processing conditions, including a melt temperature of 209.06 °C, preheating temperature of 119.48 °C, injection velocity of 60.23 mm/s, and foaming agent content of 1.55%, resulted in improved Mode I and Mode II fracture energies of 2.36 and 2.78 mJ/mm^2^, respectively. Furthermore, uniform cell distribution at the skin/core interface was found to significantly enhance the interfacial bonding quality and fracture resistance of the sandwich structures.

Lohr et al. [78] conducted an experimental analysis on the foaming behaviour of pure polyphenylene sulphide (PPS) and long glass fibre-reinforced PPS using the high-pressure foam injection moulding process. Additionally, to lower the manufacturing complexity and enable processing directly from the raw materials, an in-line extrusion (compounding) with foam injection moulding process was adopted, with N_2_ as the physical blowing agent. This combined approach allows the manufacturing of integral sandwich structures in a single stage, connected with fibre-reinforced skins and foamed core, referred to as direct thermoplastic foam injection moulding. The results of this study guide suitable process parameter ranges and clarify their influence on sandwich morphology and mechanical performance, supporting both research and industrial applications of PPS-based foam injection moulded components. The developed sandwich structures exhibit an identical skin-layer-to-core thickness ratio, while the cell size within the foamed core varies as a function of the applied delay time.

Weidmann et al. [34] reported the fabrication of mono-polymer thermoplastic sandwich panels through an in situ low-pressure injection foaming process. Unidirectional continuous glass fibre-reinforced polypropylene as skins and PP homopolymer as the foam core were employed. In the initial part, the use of blowing agents and additional nucleating agents was restricted for the in situ injection process to allow an isolated investigation of the fusion bonding behaviour between two miscible semi-crystalline skin/core polymer surfaces. The results showed that interfacial bonding was strongly governed by melt and mould temperatures. Low process temperatures led to incomplete fusion and poor stress transfer. In contrast, higher temperatures enabled full melting of the skin surface, formation of an interphase, and significantly improved interfacial strength and fracture toughness. In the second part of his study, the core was produced using different foam injection moulding variants, and the influence of both chemical (Hydrocerol^®^ ITP 822) and physical blowing agents (CO_2_) on the interfacial bonding between skins and the integral foam core was examined. The results showed that the type of blowing agent has no significant effect on interfacial fracture toughness when comparable process temperatures are applied. For both chemical and physical blowing agents-based foam cores, interfacial bonding increases with increasing melt temperature and reaches similar maximum values. However, a slight reduction in interfacial bonding at high melt temperatures was observed for chemically foamed cores, whereas physically foamed cores showed a continuous improvement. Overall, reduced interfacial bonding was primarily attributed to lower cavity pressure during foam injection moulding rather than the blowing agent type. In the final part of the study, the authors compared the varied core materials to determine the effect of particulate fillers on the foaming behaviour of the core and the resulting flexural rigidity of the sandwich structures. Therefore, the second sandwich panel with the core material of 20 wt.% talcum-filled polypropylene homopolymer (PP-T20) was foamed with the same processing conditions as the unchanged core in situ panels. The 20 wt.% talcum-filled in the PP-T20 cores reduced cell size during chemical foaming, yielding moderate density reduction and flexural rigidity compared to unfilled PP cores. For physical foaming, PP-T20 showed strong melt temperature dependence, demonstrating that fillers affect foam morphology, process sensitivity, and lightweight design performance.

Hüttl et al. [63] demonstrated that sandwich foam injection moulding enables the one-shot production of lightweight thermoplastic sandwich structures by combining short recycled carbon fibre-reinforced outer skin layers with unreinforced foamed cores. Carbon fibre-reinforced thermoplastics, including systems containing recycled fibres, were successfully processed together with foamable, material-compatible thermoplastics using chemical or physical blowing agents at supercritical conditions. Sandwich foam injection moulding of two thermoplastic materials was performed using a two-component injection moulding machine with independently controlled plasticising units, enabling precise adjustment of skin and core layer distribution. The cavity was first partially filled with the skin material, forming a solid layer at the mould wall, followed by injection of the gas-loaded core material, which displaces the molten interior until complete filling. Foaming occurs only after a pressure drop in the mould, while a needle-valve nozzle prevents premature gas release. Chemical blowing agents can be processed using standard plasticising units. In contrast, physical foaming with nitrogen was preferred for high-volume production due to its ability to achieve lower foam densities and enhanced lightweight potential. The study highlights the effective use of recycled polymers (PE/PP, PET, and PA) as core materials and shows that the resulting sandwich structures exhibit high specific stiffness and are suitable for cost-efficient, high-volume applications, particularly in the automotive sector.

In situ injection foaming is widely adopted for producing thermoplastic foams with complex three-dimensional geometries, integrated skin layers, and high dimensional accuracy. The method enables precise control of cell morphology, including cell size, distribution, and density, through careful adjustment of blowing agent content, melt temperature, cavity pressure, and injection speed [146,147,148,149]. Additionally, the process improves melt flow, reduces clamping force and cycle time, and allows the fabrication of complex geometries with good dimensional stability, making it suitable for high-volume industrial applications. Although injection foaming enables the semi-continuous production of complex three-dimensional parts with smooth surfaces and short cycle times, it has limitations compared to batch and extrusion foaming. Unlike batch foaming, the mould confines the polymer, restricting foam expansion and resulting in lower weight reduction and smaller cell growth, while slower depressurisation in batch processes allows more uniform microcellular structures. Compared to extrusion foaming, injection foaming is less suitable for producing continuous sheets or profiles and typically achieves lower throughput for long, simple geometries. Moreover, injection foaming requires precise control of gas content, temperature, and pressure to maintain uniform cell distribution, and surface imperfections such as flow lines or minor porosity are more common [150,151,152]. The process also demands more expensive equipment and tooling than both batch and extrusion foaming.

### 3.5. Welding

Welding has emerged as a recent trend for achieving skin-core fusion bonding in thermoplastic sandwich manufacturing, driven by the growing need for low-cost, high-performance structures. Unlike other fusion-bonding methods, welding enables rapid, direct molecular bonding with minimal material deformation between thermoplastic skin/core components [104,153,154]. This leads to improved interfacial strength, lower energy inputs, reduced material wastage and shorter production cycles by avoiding several interlinked processing steps. The increasing use of thermoplastics in the automotive, aerospace, and transportation sectors has further accelerated the adoption of welding-based joining methods for sandwich structures, particularly when in situ skin/core bonding or repair is necessary. Among the available welding techniques, ultrasonic welding (USW), induction welding (IW), and resistance welding (RW) have recently gained particular relevance for thermoplastic sandwich manufacturing due to their efficiency, controllability, automation capabilities, and compatibility with fibre-reinforced thermoplastics [155,156,157].

USW is an effective joining method predominantly utilised in fibre reinforced thermoplastic composites, in which low-amplitude, high-frequency vibrations are applied perpendicular to the joining surfaces to generate localised heating and achieve fusion bonding [158,159]. Its key advantages include very short welding cycles, typically from fractions of a second to a few seconds, and the absence of any additional material at the joint interface for both carbon- and glass-fibre-reinforced thermoplastic composites. The process is well-adaptable for spot welding and can be readily automated. Heat generation in USW results from interfacial friction and viscoelastic dissipation at the molecular level [159]. Unlike many other welding techniques, the heating mechanism is strongly coupled with the applied contact pressure [160]. Heat generation during the vibration phase is primarily governed by the frequency, amplitude, and applied force, while the combined effect of these parameters with vibration and solidification times determines the final weld quality [157,159,161].

**Figure 9:**
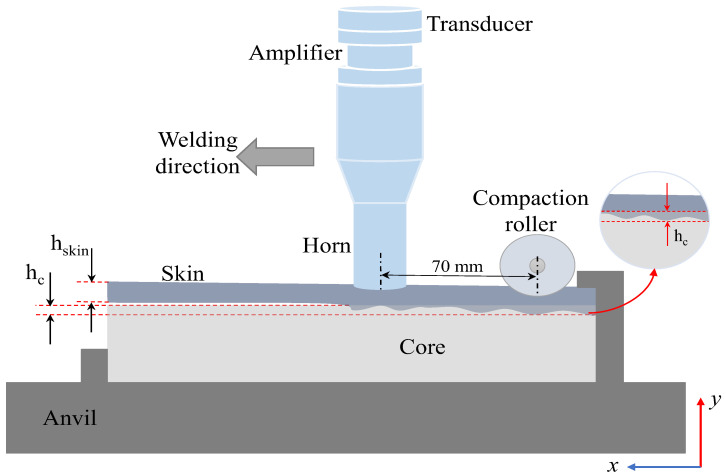
Design of a continuous ultrasonic welding setup for sandwich panels (Picture courtesy of [11]).

Research on ultrasonic welding (USW) of thermoplastic sandwich panels remains limited, with only a few studies reported. Oliveira et al. [45] implemented an alternative joining approach based on static USW that has been developed for upcycled HDPE honeycomb-based sandwich panels. The schematic representation of the continuous USW arrangement in skin/core welding is shown in Figure 9, where the skin/core stack was clamped to restrict the motion during welding. Flax fibre/poly-lactic acid (PLA) and glass fibre (GFRP)/high-density polyethylene (HDPE) skins are joined directly to an upcycled HDPE honeycomb core derived from discarded bottle caps. The resulting welding process reduced the estimated environmental footprint of bottle-cap-based panels by up to 71%, and their eco-mechanical efficiency increased up to 130% compared to equivalent structures produced using adhesive bonding. Static ultrasonic welding between the HDPE-based GFRP skins and the HDPE honeycomb core produced strong skin/core bonding, with localised delamination observed around a limited number of joints. These results showed weight-specific flexural properties higher than adhesive-bonded structures (with increases of up to 45%), while the specific energy absorption under impact loading was enhanced by up to 23% when welded joints were used. On the other hand, the weak bonding between the flax-fibre-reinforced PLA skin and the HDPE bottle-cap core reduced the bending and impact performance of the sandwich panels by up to 66% with welded joints.

Degen et al. [162] showed the fabrication of a 3D sandwich structure of polycarbonate using ultrasonic spot welding and demonstrated the flexibility in the fabrication technique. The ultrasonic spot-welding method was employed to join three stacked layers of polycarbonate sheets into a 3D sandwich structure. This single-polymer sandwich panel uses an alternating pattern of ultrasonic spot welding, with the middle layer joined to each of the outer layers. Impact testing demonstrated that the sandwich structures exhibited enhanced capability to attenuate impact loads, indicating their suitability for applications requiring effective resistance to shock and impact loading. The results indicate that the fabricated polycarbonate sandwich structures are well suited for applications demanding high strength and energy absorption at low weight. Moreover, the ease of integrating different geometrical configurations enables the design of structures with tailored mechanical performance. Additionally, multi-spot USW was also mentioned in the manufacturing of carbon fibre-reinforced polyetheretherketone (CF/PEEK) composite honeycomb core [163]. Alliyankal Vijayakumar et al. [11] developed a continuous USW approach for manufacturing mono-polymer sandwich panels, comprising PP Twintex^®^ skins and foam core. This method of manufacturing overcomes the limitations of non-welded areas and weak bonding in static USW. Mode I characterisation revealed that skin delamination and non-welded areas occur at a higher welding speed and lower horn pressure. In contrast, strong Mode I interfacial bonding toughness was achieved at 4 mm/s under 0.28 MPa (3368 J/m^2^) and at 5 mm/s under 0.39 MPa (3323 J/m^2^), exhibiting cohesive failure.

Induction welding (IW) is an efficient non-contact technique of joining thermoplastic composites, in which an alternating current in an induction coil generates a time-varying electromagnetic field that induces eddy currents in electrically conductive components (adherents) [164]. This leads to localised heating and interfacial melting or softening of the surrounding thermoplastic polymer. Pressure may then be applied during or after the heating stage to complete the welding process [155]. A complete induction welding system depends on the application, but typically consists of five main components: a radio-frequency power generator that supplies the required current and voltage to the induction coil. The second is a heating station containing the induction coil, which generates the alternating magnetic field for heating. Third, the composite workpiece to be joined, followed by a pressure application system, such as a press or vacuum bag, used to consolidate the joint during or after heating, and auxiliary equipment, including water-cooling systems, required for stable and safe operation. Heat generation at the interface of adherents occurs through two primary mechanisms: the formation of induced eddy currents in electrically conductive materials [165,166,167] and hysteresis losses in magnetic materials [168,169]. Carbon fibre-reinforced thermoplastics can be heated by induction without the need for additional materials, as the carbon fibres themselves are electrically conductive. This welding approach is known as susceptor-less welding [164,169,170]. In contrast, glass or natural-fibre reinforced thermoplastics can only be heated by induction when an additional electrically conductive susceptor material is used at the joint interface between the components to be joined. A main limitation of the susceptor-less induction heating approach is related to the formation of eddy current loops within electrically conductive materials. As the induction coil moves toward the edges of the laminate/adherent, these current loops become constricted, leading to a local increase in current density. Consequently, excessive heat generation occurs at the edges, commonly referred to as the edge effect [155,171].

**Figure 10:**
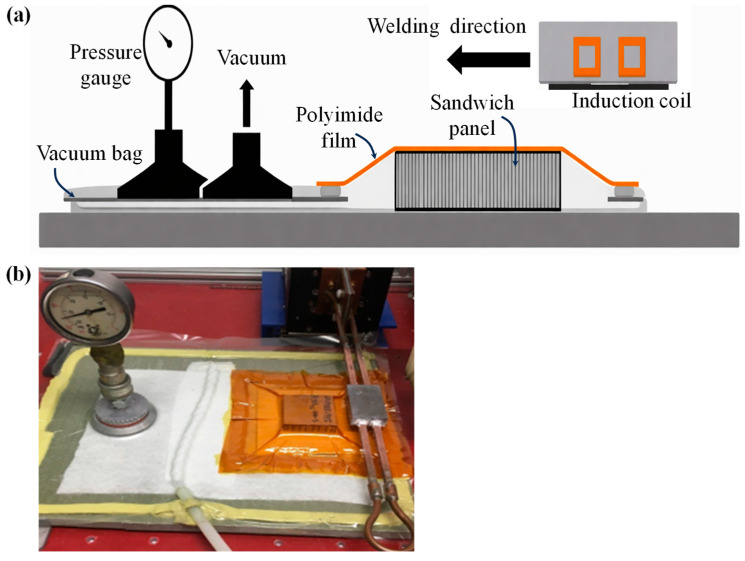
(**a**) Schematic representation and (**b**) photograph of the Vacuum-Induction welding setup. In the schematic, the black horizontal arrow indicates the relative motion between the induction coil and the sample, with the coil translation speed corresponding to the welding speed (Picture courtesy of [95]).

Most commonly, IW is employed in joining or consolidating fibre-reinforced thermoplastic composites; few studies have been reported for thermoplastic sandwich panels. A full thermoplastic sandwich panel made of glass fibre/PEEK skins with a co-consolidated 125 μm-thick PEI film and a 3D-printed PEI honeycomb core were manufactured by making use of the continuous induction heating in vacuum bagging [95]. This method uses a vacuum bag for pressure application and relies on a magnetic susceptor made of PEI and µm-sized nickel (Ni) particles (made using hot press, 0.6 mm film thickness) to generate the heat at the skin/core interface. The schematics and the vacuum-induction welding experimental setups used are presented in Figure 10. As heat is generated at the same time as the pressure is applied, this method can be considered as a one-stage process. Contrary to vacuum bagging, as the heat is generated locally and not throughout the whole structure, it is considered a welding process. Nonetheless, the use of a secondary PEI polymer with a lower welding temperature is applied to the glass/PEEK skin surfaces to avoid laminate deconsolidation and preserve skin mechanical properties (see Figure 10). Also, glass fibre reinforcement was chosen to prevent direct heating of the skin, which would occur with carbon fibres. This enables welding to be carried out using a hysteresis-losses susceptor and allows validation of this approach for induction welding of sandwich structures. Owing to the low thermal conductivity of the PEI honeycomb core, heat remains localised at the interface, thereby reducing the risk of core crushing. During this process, the second skin on the opposite side of the sandwich panel is located far from the induction coil. Given that sandwich cores are typically 0.5–1 inch thick, or even thicker, the magnetic field at the opposite skin is very weak and does not produce significant heating. Moreover, the absence of direct induction heating and the limited heat transfer through the core thickness ensure that the opposite skin remains unaffected by the welding process. Skin/core bond strengths of 5.1–5.6 MPa were measured at a lower welding speed of 0.5 mm/s, with failure occurring cohesively within the adhesive layer.

The same author, Martin et al. [172] developed a magnetic susceptor in printable form for the induction welding of glass/PEEK skin-based 3D printed PEI honeycomb core sandwich panels. The susceptor consists of Ni particles dispersed in a PEI matrix, extruded into filament form and spooled for 3D printing. PEI honeycomb core with an integrated susceptor layer of 1 mm was fabricated using a primary and secondary printing heads. The susceptor-integrated honeycomb can be welded directly to the GF/PEEK skins without additional materials. This enables welding at PEI temperatures (~280–300 °C) while avoiding deconsolidation of the PEEK laminate. Honeycomb cores with an integrated printed susceptor layer demonstrated a good printability on complex geometries. The strongest specimen failed within the core at a flatwise tensile strength of 4.6 MPa at 0.15 mm/s, indicating that the weld strength exceeded that of the core. Moreover, Martin et al. [173] utilised the same induction welding approach to assemble sandwich panels consisting of carbon fibre-reinforced PEEK skins with a co-consolidating layer of PEI film and 3D-printed PEI honeycomb core without using a susceptor. In this scenario, the skins are expected to heat through the full thickness rather than being locally heated at the joint interface, as occurs when a susceptor is employed. The use of PEI layer co-consolidated on the CF/PEEK skins permits the welding at 280–300 °C, below PEEK’s melting point, to prevent core crushing and skin deconsolidation while enabling PEI-PEI fusion joining. Flatwise tensile tests indicate that this process achieves higher skin/core strength (7 MPa at 0.7 mm/s) than previously reported methods. Fracture analysis reveals that excessively wide or narrow skins lead to non-uniform heating, affecting weld quality, highlighting the importance of optimizing skin dimensions.

**Figure 11:**
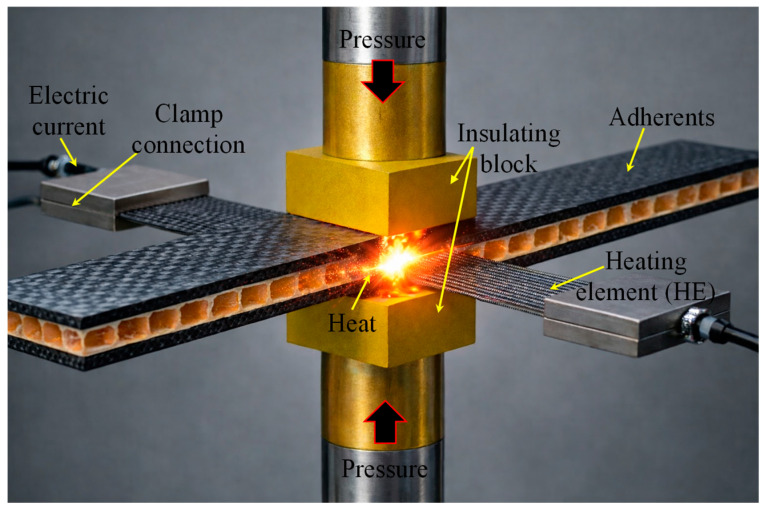
Resistance welding graphical representation.

Resistance welding (RW), also referred to as resistive implant welding, electrical-resistance fusion, or electro-fusion, is widely regarded as one of the most attractive joining techniques for fibre-reinforced thermoplastic composites [104,174,175]. A typical RW experimental setup, illustrated in Figure 11, comprises several standard components. These include the welding plies, between which the electrically resistive heating element (HE) is positioned, as well as insulating blocks to limit heat losses. Pressure is applied using a dedicated loading tool, while an electrical power supply provides the current required for heating. The assembly is completed with a clamping system to ensure proper alignment and contact, electrical wiring, and instrumentation such as voltmeters and ammeters to monitor the welding process. The process is characterized by its relative simplicity and high level of controllability, making it particularly suitable for structural applications. In this method, an electrically resistive implant (commonly referred to as a heating element, HE), typically in the form of a metallic mesh, a carbon-based element, or a conductive polymer layer, is placed between the mating surfaces of the thermoplastic composite laminates, as shown in Figure 11.

Upon the application of an electrical current, Joule heating is generated within the implant (HE) due to its electrical resistance, resulting in localised heat generation at the joint interface [154,176,177]. This localized heating promotes melting or softening of the thermoplastic matrix in the adjacent laminate plies, enabling intimate contact, molecular interdiffusion, and subsequent fusion bonding upon cooling. Similar to the susceptor used in induction welding, the conductive implant remains within the joint after welding, which can be advantageous for subsequent reprocessing. However, it may adversely affect the mechanical performance of the bond and increase both the structural weight and the risk of corrosion when metallic implants are used [174]. As the heat is generated internally at the interface, the surrounding composite structure experiences limited thermal exposure, thereby reducing the risk of fibre distortion, matrix degradation, or residual thermal stresses [153]. This type of welding is performed irrespective of the electrical or physical properties of the reinforcing material. Consequently, this technique is suitable for a wide range of fibre-reinforced polymer composites, including those reinforced with carbon, glass or natural fibre [154,174].

**Figure 12:**
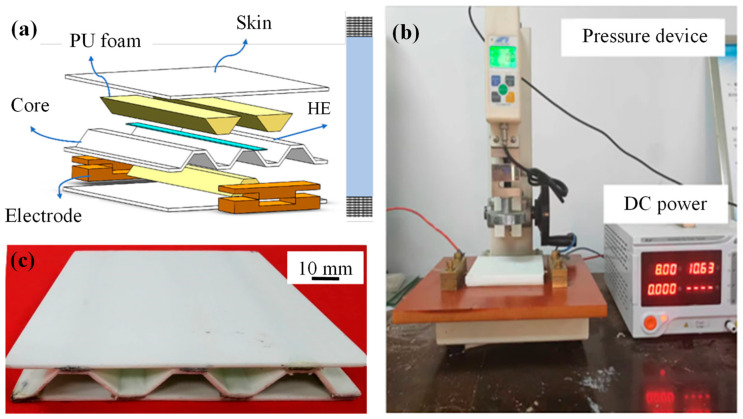
(**a**) Schematics of the resistance welding of corrugated core sandwich panels, (**b**) resistance welding device, and (**c**) resistance-welded sandwich panel (Picture courtesy [178]).

Resistance welding is well established for joining fibre-reinforced thermoplastic composites; however, its application to the manufacturing of thermoplastic sandwich structures is relatively recent, and only a single study on a thermoplastic sandwich panel has been reported using this technique to date. Zhang et al. [178] joined the corrugated cores to the skins in two ways, using hot-melt bonding (HB) and resistance welding (RW) techniques. The prepregs of glass fibre (GF)-reinforced PP were used to manufacture the skins and corrugated core by hot press. During the hot-melt bonding (HB) process, the interface between the skins and the core was softened by melting separately with a hot plate, followed by the application of pressure. Resistance welding units (see Figure 12a,b), made by sandwiching stainless steel (HE) between GF/PP prepregs, were placed at the interface of the skin and corrugated core, with a PU foam block for support. Joule heating was applied via a DC power supply under pressure, followed by 30 s pressure retention after switching off the current. The corrugated sandwich panel produced via RW is shown in Figure 12c. RW significantly enhances the skin/core interface strength in corrugated sandwich panels. Peak loads were 42% and 92% higher than those of hot-melt bonded panels in edgewise compression and three-point bending tests, respectively. Initial defects in hot-melt bonding accelerate crack propagation, while resistance welding ensures an intact interface. These results demonstrate that resistance welding is a viable skin/core fusion bonding technique for thermoplastic sandwich structures, with strong potential for aerospace applications.

Although welding-assisted bonding techniques provide significant advantages for rapid and adhesive-free assembly, several approaches remain at the developmental or pilot scale. Further work is required to improve process robustness, joint consistency, and scalability for large structural sandwich components.

### 3.6. Automated Fibre Placement

**Figure 13:**
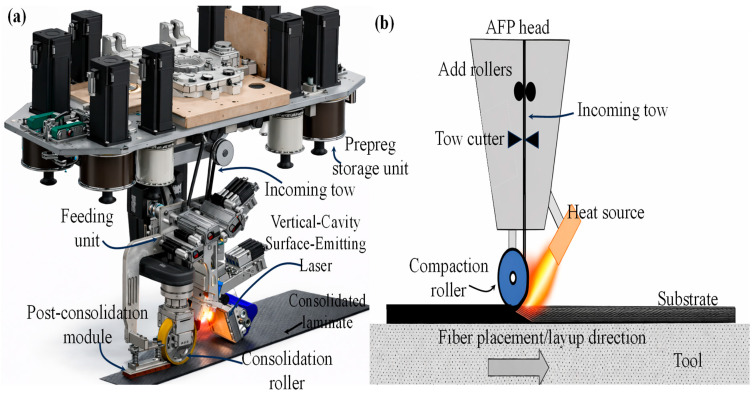
(**a**) The thermoplastic AFP deposition head developed for the sandwich panels (Picture courtesy of [185]), and (**b**) a schematic illustration of the AFP head (Picture courtesy of [182]).

Automated fibre placement (AFP) is an advanced manufacturing technology widely used for fabricating high-performance composite structures, particularly in the aerospace sector [179,180,181]. The method of manufacturing thermoplastic sandwich panels using AFP is derived from the consolidation of fibre-reinforced thermoplastic laminates using AFP. The AFP process employs a gantry or robotic system equipped with a fibre-placement head that deposits multiple composite tows onto a tool surface [182]. To facilitate understanding of the complex AFP head, a simplified schematic and the AFP head unit developed by the Institute of Production Engineering and Machine Tools, Germany, are shown in Figure 13. The AFP head encompasses key components such as the add rollers, tool, compaction rollers, tow cutter, and heat source (see Figure 13).

Bonding between the incoming tows and the substrate is achieved through controlled heating sources, such as lasers, infrared heaters, or hot gas torches, compaction, and tensioning [183]. Multiple tows form a course, courses are combined to produce a ply, and successive plies build up the laminate. Process parameters such as placement speed, heating power, consolidation force, and tow tension play a critical role in determining the quality of interlaminar bonding, void content, and residual stresses [182,183]. A series of parallel tows forms a course; multiple courses constitute a ply, and stacking of plies with varying fibre orientations results in a laminate with tailored mechanical properties. The ability to perform in situ consolidation significantly reduces manufacturing time, allowing for the near-net-shape production of complex structures [184].

Conventional laser systems are unsuitable for sandwich manufacturing due to their uniform radiation profiles. The advanced vertical cavity surface emitting laser (VCSEL) systems were developed by Denkena et al. [185]. This enabled zone-specific temperature control, which ensures controlled melting at the skin/core interface. The developed laser-assisted thermoplastic AFP head with the VCSEL system is illustrated in Figure 13a. In this study, thermoplastic CF/PEEK prepreg tapes were deposited on a PEI closed-cell foam core for the production of sandwich panels to achieve fusion bonding. The findings indicate that a cohesive interface is achieved between the placed tapes and the foam core. Nevertheless, the cellular morphology in the area of the deposited tapes is influenced by the applied processing conditions, with deformation becoming more pronounced at elevated deposition temperatures. The results show that foam deformation can be reduced by using a short contact time and a high processing temperature, while promoting the formation of the thin molten layer required for cohesive bonding. Under these conditions, the internal structure of the foam core remains largely unchanged. Additionally, increasing the joining temperature significantly improves bonding strength in AFP-manufactured thermoplastic sandwich interfaces. The specific peel force increases from 0.79 N/mm at 280 °C to 1.15 N/mm at 360 °C, corresponding to an improvement of approximately 45%.

Similarly, Siemen et al. [186] investigated the use of AFP for thermoset sandwich structures using different prepregs and adhesive films in combination with a Nomex^®^ honeycomb core (96 kg/m^3^, 3.2 mm cell size). They identified the bridging effect as a key defect that reduces interfacial quality through local debonding. The study showed that material tackiness, measured via probe tack tests, strongly influences bridging formation and can be used as a screening parameter for AFP suitability. In addition, path-planning parameters, especially lay-up direction and resolution, significantly reduce bridging, with reported reductions in unbonded area by more than a factor of five. AFP process parameters such as lay-up velocity and lamp power were also found to have a statistically significant effect. Overall, combined optimisation of material, process, and path parameters reduced bridging by up to a factor of 6.9, improving the quality of sandwich manufacturing.

**Figure 14:**
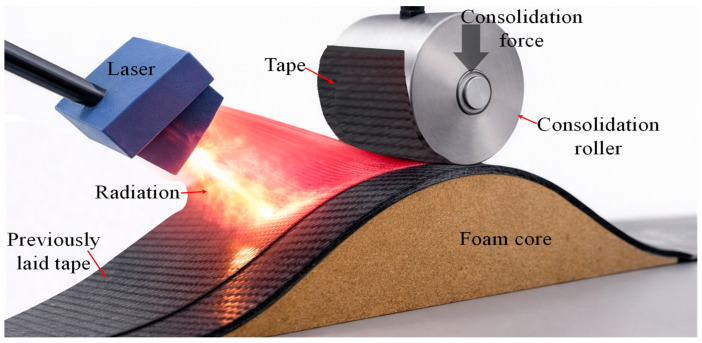
Unconventional aircraft panel stiffener manufacturing method through thermoplastic automated fibre placement (Picture courtesy of [185]).

AFP shows great potential for the production of geometrically complex, automation and customized sandwich structures (see Figure 14), as it enables precise, programmable placement of multiple narrow tows along variable paths on complex geometries, reducing tooling complexity and allowing high levels of design flexibility [182,187,188]. The application of AFP in thermoplastic sandwich manufacturing is still largely limited to laboratory-scale and specialised industrial demonstrations. Challenges associated with processing complexity, equipment cost, and integration with complex core geometries continue to limit widespread industrial adoption.

### 3.7. Additive Manufacturing

Additive manufacturing (AM) in thermoplastics refers to a family of layer-by-layer deposition techniques in which thermoplastic polymers are processed in the molten or softened state and solidify upon cooling to form a three-dimensional part directly from a digital model [189,190,191]. Unlike thermoset-based manufacturing routes, thermoplastic AM relies on physical melting rather than chemical curing, which enables remelting, fusion bonding, repair, and recycling [192]. This characteristic makes thermoplastic AM particularly attractive for lightweight structural applications where joining, multifunctionality, and sustainability are important design drivers.

Several AM technologies are used for processing thermoplastics, with material extrusion, powder bed fusion, material jetting, and large-scale directed energy deposition being the most relevant [193,194,195]. Material extrusion processes, such as fused filament fabrication (FFF) or fused deposition modelling (FDM) and foam additive manufacturing (FAM), are the most widespread due to their simplicity and low cost. In these processes, a thermoplastic filament or pellet feedstock is heated and extruded through a nozzle, depositing material in layers. Commonly processed materials include PLA, ABS, PA, and PETG, as well as high-performance polymers such as PEEK, PEI, and PPS, sometimes reinforced with short fibres. Powder bed fusion techniques, such as selective laser sintering (SLS) and multi-jet fusion (MJF), rely on the selective fusion of thermoplastic powders using a laser or thermal source, offering improved geometric freedom and more isotropic mechanical properties compared to material extrusion. Material jetting processes deposit droplets of molten or solution-based thermoplastics and are primarily used for high-resolution prototyping rather than for load-bearing structures. For large components, robotic or gantry-based extrusion systems, often referred to as thermoplastic directed energy deposition, allow high deposition rates and the manufacture of large structural parts.

**Figure 15:**
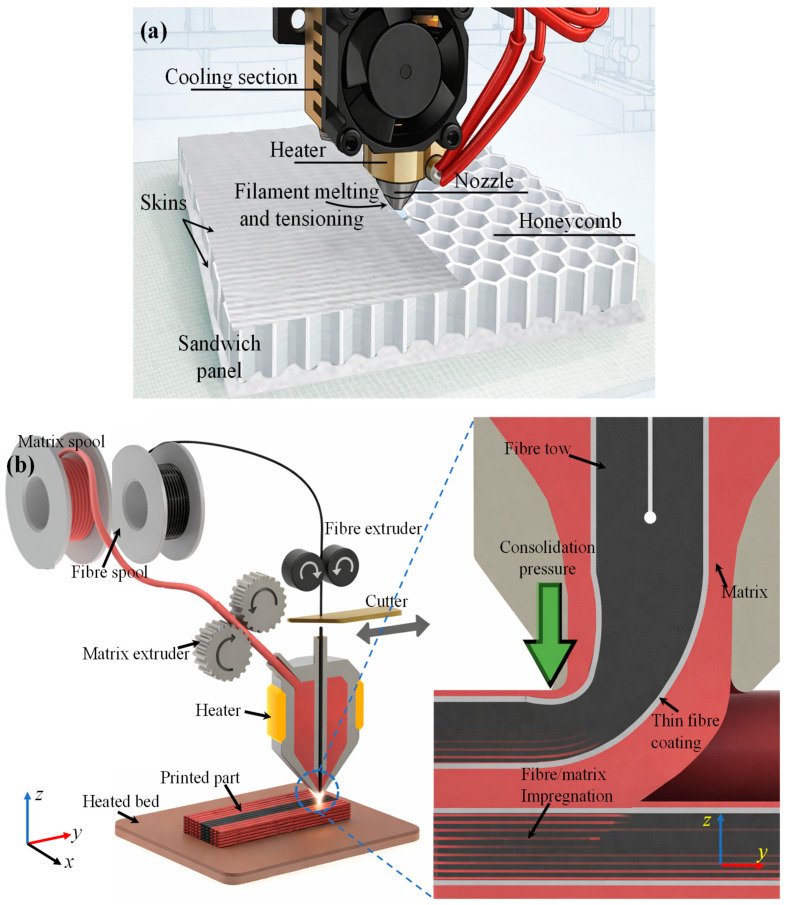
Schematics of filament-based printing in sandwich structures using (**a**) extrusion (single filament) and (**b**) co-extrusion processes (Picture (**b**) courtesy of [51]) (multi-material: two filaments).

**Figure 16:**
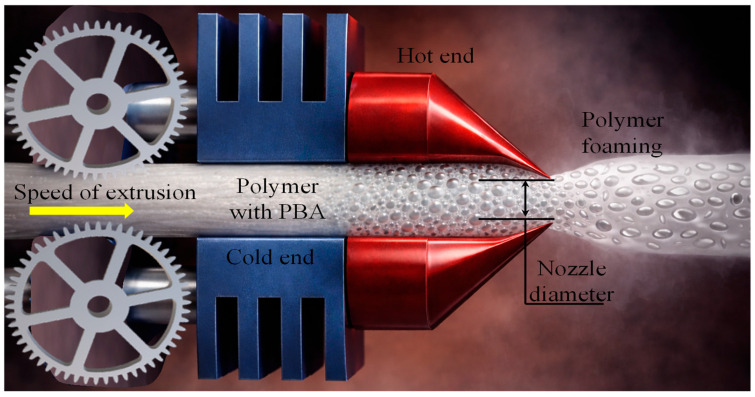
Outline of the extrusion process in foam additive manufacturing (Adapted from [48]).

In the context of sandwich panel manufacturing, additive manufacturing introduces an ideal shift compared to traditional fabrication routes based on pre-manufactured cores and adhesive bonding. Conventional sandwich panels typically consist of thin skins bonded to a honeycomb or foam core using structural adhesives, which limits geometric customization, introduces additional weight, and can lead to interfacial failure modes. Thermoplastic additive manufacturing enables the direct fabrication of cellular or lattice cores with tailored geometry, density, and mechanical response, including honeycomb, foam, corrugated, and graded lattice architectures. In filament-based material extrusion processes (Figure 15a), a continuous thermoplastic filament is fed by drive gears into a heated nozzle, where it is melted and extruded as a semi-molten strand that is deposited layer-by-layer following a programmed toolpath and solidifies upon cooling [196,197]. A dedicated cooling section maintains a sharp thermal gradient between the cold and hot ends, ensuring stable filament feeding, dimensional accuracy, and rapid solidification of the deposited material, which is particularly critical for preserving cellular morphology in foamed structures (see Figure 16). This controlled deposition allows the integrated manufacturing of sandwich structures, in which the bottom skin is printed first, followed by the direct deposition of the core, and subsequently the printing or fusion bonding of the top skin within a single, automated process, as depicted in Figure 15a.

Several monopolymer thermoplastic sandwich panels manufactured using AM are shown in Table 1. Bharath et al. [46] used HDPE filament to fabricate the sandwich skins, while the cores were produced from HDPE-based syntactic foam filaments reinforced with glass micro balloons (GMB). Filaments containing 20, 40, and 60 vol.% GMB were prepared using optimized blending and extrusion parameters and printed using a commercial FFF system. The sandwich panels were manufactured in a single printing sequence by consecutively depositing the skin/core/skin layers. Flexural characterisation revealed that increasing GMB content enhanced the specific stiffness and strength of the sandwich composites, with the highest performance achieved for the core containing 60 vol.% GMB. The flexural strength of sandwich panels with 20, 40, and 60 vol.% GMB is 1.05, 1.22, and 1.35 times higher, respectively, compared to the corresponding 20, 40, and 60 vol.% GMB cores. Sandwich panels with 20 vol.% GMB (SH20) remained intact up to 10% strain, while SH40 and SH60 fractured in a brittle manner due to the higher glass microballoon content. Additionally, no shear failure was observed in the additively manufactured sandwich constructions.

Monolithic sandwich structures were manufactured using a continuous carbon fibre 3D printer operating under continuous-line conditions by Sugiyama et al. [49]. Due to the absence of a cutting mechanism, the skins and core were printed in a single uninterrupted process. The skins were produced by depositing continuous carbon fibre-reinforced PLA filaments in linear paths with alternating 0°/90° stacking, while the core was generated with the same filaments using periodic unit-cell-based geometries, enabling honeycomb, rhomboid, rectangular, and circular core configurations. Support-free printing of wide bridge regions was achieved by exploiting the tensile stiffness of the continuous carbon fibre, and abutment features were incorporated to maintain fibre tension during top-skin deposition. As a result, fully integrated PLA sandwich panels with continuous fibre reinforcement throughout the skins and core were obtained. The results of three-point bending tests indicated that the core geometry largely governs the mechanical properties of these sandwich structures. Arslan et al. [67] investigated the flexural properties of sandwich structures fabricated using high-temperature thermoplastic composite filament-extrusion 3D printing, based on recycled PEI and PPS matrices. Various PEI and PPS composite filament formulations incorporating recycled carbon fibre (rCF) and thermal black (TB) fillers were made to fabricate sandwich panels in a single stage. The sandwich panel skins were printed in the *xy* plane with a ±40° raster orientation relative to the *x*-axis and a 100% infill density. Sandwich panels fabricated with the developed filaments exhibited flexural performance comparable to that of printed with commercial materials (15 wt.% short CF/PEI and 15 wt.% short CF/PPS), sustaining bending loads of up to 3 kN with a mass of approximately 50 g. PEI-based panels (density 384 kg/m^3^) achieved core shear strengths and moduli of up to 1.4 MPa and 80 MPa, respectively, while PPS-based panels (density 386 kg/m^3^) reached values of 1.7 MPa and 128 MPa. Notably, the core shear modulus of panels printed with the developed filaments was approximately twice that of panels produced using commercial filaments. Additionally, Faidallah et al. [53] extruded single material PLA filament for AM manufacturing of sandwich panels for core geometries such as honeycomb and rombus. Sandwich panels with rhombus core showed superior mechanical performance, with tensile, bending, and compression strengths 15.3%, 39.8%, and 35.1% higher than those of the honeycomb core, respectively. A similar manufacturing was reported by Zaharia et al. [54]. Biodegradable PLA/PHA sandwich structures were fabricated with honeycomb, diamond-celled, and corrugated cores in a single part. The diamond-celled core showed the highest compression and three-point bending strengths, while the corrugated core exhibited the highest tensile strength.

Verville et al. [51] developed a fibre-reinforced AM infrastructure that produces multi-material sandwich panels in a single manufacturing step by co-extrusion of continuous carbon fibre-reinforced PLA, wherein the thermoplastic matrix and continuous fibre reinforcement are combined in situ during extrusion. Schematics of the co-extrusion process are illustrated in Figure 15b. The hotend melts the thermoplastic filament, while the central capillary tube feeds continuous fibre tows to the nozzle tip. The fibres, together with their TPU coating, are mixed with the molten PLA and extruded through a 1.2 mm nozzle. The co-extruded composite is deposited layer by layer, consolidated by the heated nozzle, and cooled to retain shape as a sandwich panel. Fibre tension creates a matrix-rich region that improves impregnation, and a final thin matrix-only layer ensures full fibre embedding. Similarly, Feng et al. [50] manufactured 3D-printed continuous carbon fibre-reinforced PLA honeycomb sandwich structures with fibre-adjacent and fibre-interleaved core using the FFF co-extrusion method. Both PLA filament and continuous carbon fibre bundles were fed into a heated nozzle maintained at 240 °C, where the fibres were impregnated by molten PLA and extruded to form solidified layers. This was deposited sequentially on the build platform layer by layer, following a programmed nozzle toolpath. Printing parameters, including a nozzle diameter of 1.0 mm, printing speed of 4.0 mm/s, and hatch spacing of 0.8 mm, were optimised experimentally. Integrated sandwich structures were produced by sequentially printing the bottom skin, the continuous-fibre-reinforced honeycomb core (with fibre-adjacent and fibre-interleaved cores), and the top skin in a single process, with fibre tension maintained to enable support-free fabrication of the core. The results demonstrated that the sandwich panels with the fibre-interleaved core enhanced the mechanical properties (i.e., 44% higher in elastic modulus and 119% higher in strength), due to their design stability and high load-bearing capacity.

Another filament-based extrusion approach uses multiple printer heads or extruders, each dedicated to a different material. In this method, one print head extrudes the skin material (typically a fibre-reinforced thermoplastic), while another deposits the core material (often a lightweight or patterned structure). The heads operate in a coordinated manner, allowing skins and core to be printed sequentially or alternately within the same build. Vellaisamy et al. [64] used an ultra-high-strength continuous carbon fibre reinforced in nylon and an ONYX-FR matrix filament to fabricate a single integrated honeycomb sandwich structure. ONYX-FR, used as the core material, is a commercially available flame-retardant nylon reinforced with short carbon fibres. The continuous carbon fibre/nylon and ONYX-FR filaments are processed through separate extruders and melted in independent nozzles at 272 °C. The molten material is deposited layer by layer onto the build platform with a layer thickness of 0.125 mm, following a fibre orientation sequence of 0/±45/90/−45. The experimental results indicate that the sandwich configuration with 3.2 mm skin thickness and a 12.7 mm honeycomb cell size demonstrates superior energy absorption under both edgewise and flatwise compression loading, achieving increases of 37% and 96%, respectively, compared with conventional Al honeycomb/CFRP sandwich panels.

Moreover, in situ foaming along with simultaneous skin/core bonding using foam additive manufacturing (FAM) paved the path for a novel in situ sandwich panel fabrication with foamed structures as the core. In this method, the filament is pretreated with a physical blowing agent (PBA). As illustrated in Figure 16, the PBA expands during filament extrusion under heat and pressure, forming bubbles within the polymer while preserving its original chemical structure, in contrast to chemical blowing agents (CBAs) [192,198]. This characteristic provides lifecycle benefits, particularly by improving the recyclability of the polymer. Accurate control of printing parameters, including absorption pressure and duration, desorption time, temperature, and extrusion speed, enables the fabrication of foams with tailored properties.

Epasto et al. [48] examined the flexural behaviour of PLA-based sandwich panels produced through the FAM process. This approach enables the fabrication of complex foamed architectures with controlled morphology and density gradients using a PBA CO_2_. It allows the design of single-material components with locally tailored densities and mechanical properties, including functionally graded structures, through appropriate control of process parameters. Bulk PLA layers were printed as the top and bottom skins, while the foamed PLA with closed-cell structures were printed as the infill using the same parameters in a single, fully automated 3D printing process. Conventional honeycomb sandwich panels were also fabricated entirely from bulk PLA, with the cores produced using different infill patterns and unfoamed bulk PLA skins. A comparative analysis of foamed and bulk specimens demonstrated that, in applications where energy absorption is critical, foamed structures exhibited superior average performance compared to honeycomb specimens. Likewise, the same authors, Rizzo et al. [47], studied the low-velocity impact response of PLA sandwich panels manufactured under the FAM process. The results indicated that foamed structures with tailored core densities possess notably high impact strength and distinct energy absorption characteristics relative to traditional honeycomb configurations. Failure analysis revealed that energy absorption in foamed specimens was enhanced by core indentation and foam cell collapse, with specific energy absorption reaching approximately 0.06 J m^3^/kg for low-density.

These integrated approaches of thermoplastic AM include high design freedom, efficient material usage, reduced tooling requirements, and the ability to fabricate complex geometries such as lattice or cellular structures that are difficult or impossible with conventional methods. Consequently, thermoplastic AM is commonly used for functional prototyping, customised components, tooling, aerospace interior and secondary structures, automotive lightweight parts, and energy-absorbing cellular architectures. Despite the advantages of AM of thermoplastic sandwich structures, most current studies remain limited to prototype-scale structures and experimental validation. Issues related to manufacturing speed, dimensional consistency, large-scale production capability, and industrial qualification must still be addressed before broader commercial implementation can be achieved.

### 3.8. Thermoforming

**Figure 17:**
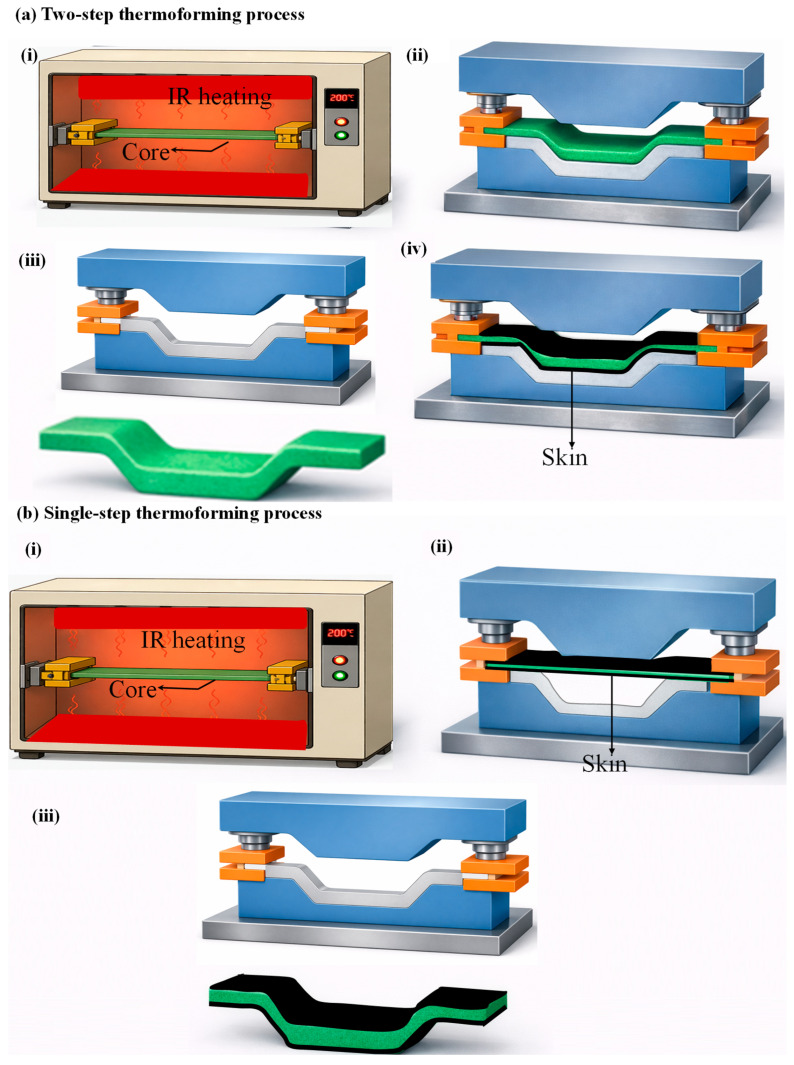
Schematics of the (**a**) two-step thermoforming process: (**i**) heating foam inside the chamber; (**ii**) forming; (**iii**) thermoformed foam; (**iv**) skin/formed-core joining, and (**b**) single-step thermoforming process: (**i**) heating skin/core inside the chamber; (**ii**) forming; (**iii**) thermoformed sandwich panel.

**Figure 18:**
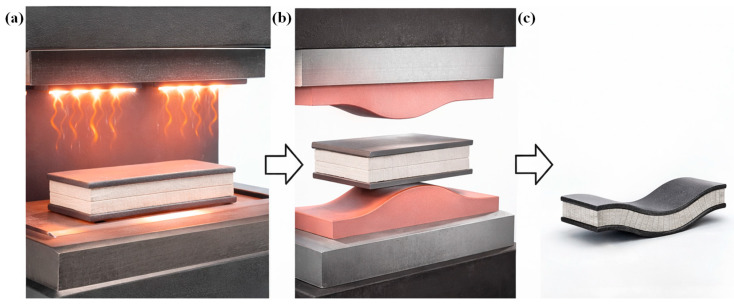
Combined thermoforming method: (**a**) heating of the skin/core stack, (**b**) fusion bonding and forming in a hot press and (**c**) thermoformed sandwich panel.

Thermoplastic sandwich structures with complex three-dimensional geometries can be produced using two main thermoforming approaches, as schematized in Figure 17. In the first approach, the foam core is initially thermoformed into the desired shape and subsequently bonded to the skins in a separate joining step known as a two-step process or non-isothermal thermoforming, as described in Figure 17a. Alternatively, a single-step or iso-thermal process can be employed in which both the foam core and the skin layers are thermoformed simultaneously, enabling direct consolidation into the final geometry, as shown in Figure 17b. The single-step method can reduce manufacturing time and improve interfacial bonding, while the multi-step approach offers greater flexibility in material selection and process control [4,124]. Moreover, when the skins and core are made from the same thermoplastic polymer, a fusion bond can form, further improving bond strength [66]. In contrast to these thermoforming approaches, a third method was developed by combining the single-step and two-step processes and is illustrated in Figure 18. This combined thermoforming process integrates the advantages of both approaches while minimising their respective limitations. Also, this method enables a higher temperature gradient required for adequate bonding between the sandwich components. This section provides a detailed discussion of these two variants of thermoforming. In the two-step thermoforming process, the foam core is initially heated to its softening temperature, as illustrated in Figure 17(a(i)). Once softened, the core is shaped either by compression moulding within the heating environment or by rapidly transferring it to a separate forming mould, as shown in Figure 17(a(ii)). Forming pressure is typically applied using a matched two-part mould or a vacuum bag to ensure accurate shaping. After forming, the foam core is cooled to retain the imposed geometry and then removed from the mould (Figure 17(a(iii)). The resulting thermoformed foam core can subsequently be combined with skins to manufacture sandwich structures with complex geometries using secondary processes such as resin infusion, vacuum bag consolidation, wet pressing, compression or resin transfer moulding (Figure 17(a(iv)).

Thermoforming requires a carefully controlled temperature window. Foam cores that are too cold may rupture or spring back, while excessive heat can cause warping, swelling, cell collapse, or thermal degradation [68,69,199]. Similarly, thermoplastic skins may be too rigid or poorly bond to the core at low temperatures, whereas high temperatures can lead to thermal degradation, deconsolidation, and increased void content [23,124]. Typically, foam cores are heated slightly below their glass transition temperature (T_g_), while skins are heated above their T_g_. When the forming temperature windows of the skins and foam core do not overlap, a two-stage heating strategy is adopted. Coombs [199] thermoformed 25.4 mm-thick Divinycell F50 PES foam sheets into 90° bends with inner radii of 25 mm and 50 mm, using an environmental chamber heated to 200 °C. Curved sandwich panels were then fabricated by bonding composite skins to the thermoformed foam bends: thermoset epoxy/flax skins were attached via resin infusion, while thermoplastic PP/flax skins were attached using vacuum bagging. The study revealed that the epoxy resin penetrated the upper layer of the foam’s open cells, creating a strong bond between the core and skins. In contrast, the high viscosity of molten PP prevented it from infiltrating the foam’s open cells, resulting in weaker interfacial bonding. Similarly, Hu et al. [74,75] manufactured a 3D sandwich structure with lattice or truss cores fabricated by repeated thermoforming using the continuous carbon fibre-reinforced thermoplastic PEEK. The lattice cores were produced via a hot-stamping process that exploits the repeated thermoformability of carbon fibre/PEEK. Initially, composite laminates were manufactured according to a predefined prepreg stacking sequence and subsequently waterjet-cut into trapezoidal grids. These grids were placed in release-agent-coated moulds and hot-pressed at 380 °C to fully soften the PEEK matrix and avoid fibre damage during forming. After controlled cooling to room temperature, the lattice cores were obtained. An in situ hot-pressing joining technique was employed to assemble the lattice structure, relying solely on the molten PEEK matrix without additional bonding materials, while steel padding blocks and limit fixtures were used to prevent core crushing and ensure dimensional stability.

In the single-step method, a sandwich stack, comprising thermoplastic composite skins and a foam core, is first heated to a temperature above the softening point of the skins while avoiding collapse of the core (Figure 17(b(i))). The heated panel is then placed in a mould, where pressure is applied to conform both the skins and the core to the desired geometry (Figure 17(b(ii))). During this step, the softened thermoplastic skins partially flow into the open cells of the foam, creating a strong mechanical and thermal bond at the interface. Finally, the panel is cooled under pressure, resulting in a fully formed and consolidated structure, as shown in Figure 17(b(iii)). Using this approach, a sandwich structure with a complex 3D geometry can be fabricated in a single operation, eliminating the need for a separate step to bond the core and skins. Polymer films can also be introduced between the skins and the core to enhance interfacial adhesion [4,124]. However, the processing requirements for one-step manufacturing are more stringent. The skins require sufficient pressure to achieve consolidation, while excessive pressure can cause the foam cells to collapse. Therefore, the forming pressure must be carefully controlled: it should exceed the minimum pressure needed to consolidate the skins but remain below the compressive strength of the foam core [68].

For certain skin/core combinations, the forming temperature windows of the core and skins may overlap, allowing thermoforming with a single-step heating. For example, Breuer et al. [200] produced a sandwich hemisphere using glass-fabric skins pre-impregnated with PA (melting temperature: 178 °C) and a PMI foam core (softening, T_g_, at 180 °C). Temperatures above 190 °C caused undesired post-foaming and expansion of the PMI foam, while temperatures below 180 °C risked foam fracture. For the skins, temperatures above 260 °C led to oxidation and deconsolidation, whereas temperatures below 185 °C produced dimensional defects. Consequently, the sandwich stack was heated to 260 °C to achieve proper forming. At an interface temperature of 185 °C, the stack was transferred to the mould for forming. Kabala et al. [201] adopted a single-step thermoforming using glass fabric-reinforced PA6 skins and a melamine soft-elastic foam core. To achieve adequate formability, the skins are heated above the melting temperature of the PA6 matrix (220 °C). Before forming, the semi-finished sandwich panel is heated to 260 °C to offset heat losses during transfer to the mould. The forming tool is maintained at 110 °C, which lies within the recommended range of 50–150 °C below the melting temperature of the thermoplastic in the skins. The heated sandwich is then formed in the tool and held for 20 s to allow cooling to the tool temperature and to prevent post-forming distortion.

A third combined thermoforming approach, combining two-step and one-step methods, was adopted by Rozant et al. [68,69] to overcome the inadequacies of obtaining the required temperature profile with one-step and two-step methods. In this method, three-dimensional sandwich components were produced by thermoforming of a PEI foam core combined with glass fibre-reinforced PEI skins using a combined thermoforming approach or a two-stage heating method, as shown in Figure 18. The skins and core were first bonded using an epoxy film and subsequently thermoformed. The PEI foam core was heated to 165–185 °C (<T_g_), while the skins required temperatures above 280 °C (>T_g_). Accordingly, the skin/core stack was first heated to 165 °C to allow thermal equilibration within the foam and then transferred to a second heating stage at 320 °C to raise the skin temperature. Once the skins reached 320 °C, the stack was placed in the mould for forming, enabling the successful manufacture of double-curved sandwich structures. A forming pressure in the range of 0.03–0.11 MPa was found to be suitable for both components. Thus, in this strategy, the entire sandwich structure is first heated between hot plates to a temperature within the foam’s processing window (Figure 18a), and subsequently transferred to a second heating stage to reach the temperatures required for forming the skins (Figure 18b). During the second stage, the skins are preferentially heated, and finally, 3D thermoformed panels were demoulded (Figure 18c).

Latsuzbaya et al. [66] developed a combined thermoforming process for monopolymer thermoplastic sandwich panels, enabling defect-free deformation of glass fibre-reinforced skins by forming at temperatures above the polymer softening point. Polycarbonate (PC) was used as the matrix material for both the skins and the honeycomb core. In this process, the skins and core were mounted in a holding frame and simultaneously preheated by infrared radiation. To avoid melting of the core during heating, it was spaced from the face sheets using a spring system, while the skins were manufactured wider than the core to limit overheating. Once the skins reached the required forming temperature, the assembly was transferred to a hot-press mould maintained below the polymer softening temperature, where forming and fusion bonding were completed.

Similarly, Minupala et al. [28] developed a novel thermoplastic sandwich thermoforming approach, also known as a multi-stage thermoforming technique, to reduce cycle times, thereby extending its applicability into the automotive and other transport industries. This is achieved using a monopolymer sandwich structure composed of continuous glass fibre-reinforced PP skins and a PP honeycomb core. This method enables the conversion of semi-finished thermoplastic sandwich panels into ready-to-use lightweight components. This process is designed to allow honeycomb-core sandwich laminates to conform to complex mould geometries without face-sheet wrinkling or core collapse. The manufacturing route comprises three main steps: infrared heating of the semi-finished laminate, thermoforming into the desired shape, and subsequent injection moulding of the component edges. Thermoforming is carried out in a two-sided press tool in three successive stages. First, the sandwich is formed in regions where the core must remain intact. The press is then briefly halted to allow cooling and solidification of the skin matrix through tool contact. Finally, the tool fully closes to compact the sandwich edges, which seals the core, enables integration of functional elements, and forms a transition zone to the over-moulded edges.

## 4. Conclusions

A comprehensive comparison of conventional and emerging thermoplastic sandwich manufacturing approaches is presented, highlighting recent advancements and the integration of novel processing strategies to improve bond quality and process efficiency. The discussion has covered various skin/core bonding strategies, highlighting how interfacial compatibility plays a critical role in structural performance, manufacturability, and recyclability. Adhesive bonding uses a supplementary material to join the skin and the core, thereby keeping their original structure unchanged during joining; no physical change (e.g., melting) or chemical change (e.g., curing) occurs. Nonetheless, this method is often time-consuming and labour-intensive, and it can lead to a weak skin/core bond. On the other hand, fusion bonding has emerged as a promising short-cycle bonding method for monomaterial thermoplastic sandwiches due to its potential to achieve strong interfacial adhesion without adhesives, thereby improving recyclability, repairability, and structural integrity.

A range of skin/core fusion bonding techniques in thermoplastic sandwich manufacturing has been critically examined, including compression moulding, vacuum bagging, double-belt lamination, in situ foaming, welding, automated fibre placement, additive manufacturing, and thermoforming. Each process offers different advantages in terms of production rate, geometric flexibility, cost, and control over consolidation. Among these, in situ foaming and additive manufacturing are distinct, as they involve the simultaneous formation of the core and bonding to the skins, rather than joining pre-formed skin-core components. Both double-belt lamination and compression moulding provide comparable sandwich panel bonding performance, while differing mainly in automation capability versus process control precision. However, a common challenge across all techniques remains the control of temperature distribution, consolidation quality, and interfacial bonding. These are strongly influenced by the primary factors such as heat transfer, processing parameters and material behaviour (e.g., core cell collapse or skin distortion) during heating and cooling. Therefore, careful optimisation of the manufacturing conditions is essential to ensure improved skin/core adhesion in sandwich panels.

Among conventional manufacturing routes, a one-stage non-isothermal compression moulding technique has emerged to overcome processing limitations associated with traditional multi-step sandwich fabrication, particularly those related to separate core production and secondary bonding. A modified pressure-induced batch foaming approach has been introduced to enable in situ core formation, promoting improved skin/core integration and structural continuity. In situ sandwich panels were manufactured through an extrusion process in foam additive manufacturing or by using a single or multi-head fused filament deposition method. Additionally, a combined thermoforming approach was developed to overcome the difficulties in achieving the required temperature profile with one-step and two-step thermoforming methods. Therefore, these manufacturing techniques open new possibilities for producing complex geometries, tailored architectures, and lightweight structural components. Sandwich manufacturing has also been simplified through welding and automated fibre placement techniques, which reduce cycle times and eliminate the need for complex mould geometries.

Despite these advances, several opportunities remain for further development of thermoplastic sandwich manufacturing technologies. Emerging manufacturing technologies such as AM, welding-based methods, and AFP show strong potential for integrated thermoplastic sandwich structures. However, many of these approaches are still at the laboratory or pilot stage, and further work is required to address scalability, process reliability, and industrial implementation challenges. Moreover, future research may focus on novel integrated manufacturing strategies that combine multiple processing steps into a single operation. For example, in situ skin consolidation, foaming, and skin/core bonding could be achieved simultaneously using a modified pressure-induced batch foaming process, enabling improved interfacial integration and reduced manufacturing complexity. Continuous induction welding of sandwich panels may also be explored without vacuum bagging for consolidation, with alternative consolidation methods, such as roller-assisted pressure, that could improve process efficiency and enable continuous production. In addition, additive manufacturing approaches offer promising opportunities for further innovation. Direct printing of thermoplastic skins onto a pre-foamed core could enable in situ skin consolidation and skin/core bonding, eliminating secondary joining steps. Similarly, extrusion-based foaming using foam additive manufacturing into pre-consolidated skins could enable the direct formation of the foam core within the sandwich structure, thereby improving structural integration. Moreover, further research is required to improve the control of processing conditions and interfacial bonding mechanisms during manufacturing. In particular, a deeper understanding of heat transfer behaviour, bonding/consolidation dynamics, and material response during processing is essential to ensure consistent bonding quality and structural reliability. These emerging concepts highlight the potential for highly integrated and automated manufacturing routes that could further simplify sandwich panel production while improving bonding quality and structural performance.

## Figures and Tables

**Table 2:** Summary of manufacturing methods for thermoplastic sandwich structures.

Manufacturing Routes	Advantages	Disadvantages	Production Volume
Compression moulding (CM)	Short cycle times; high automation potential; low part cost at medium-to-high volumes; high fibre volume fraction; low void content; good surface finish and dimensional control; strong core/skin bonding	High initial tooling and press cost; limited part size for large presses; risk of core crushing if pressure is not controlled; not economical for very low volumes	High (medium to mass production)
Vacuum bagging	Low tooling cost; good consolidation; adaptable to complex geometries; suitable for prototyping and small batches	Low pressure compared to CM; long cycle times; operator dependent; low throughput	Low (prototyping, one-offs)
Double-belt lamination	Continuous process; very high productivity; uniform thickness; excellent surface quality; suitable for large flat panels; high automation potential	Very high capital investment; limited to flat or gently curved panels; limited core architectures; less flexible for complex shapes	Very high (mass production)
In situ foaming	Excellent skin/core bonding; low material waste; potential for continuous or semi-continuous production; short cycle times, highly flexible for complex shapes	Difficult process control; non-uniform cell size or risk of structural collapse; property scatter; tooling/process development cost; moderate automation	Medium to high (medium-scale production)
Welding	Fast joining; localised heating; suitable for automation; repairable joints; minimal surface preparation; low energy consumption; restricts core collapse; mould-free; short cycle times	Limited joint area; thermal degradation risk; residual stresses; equipment cost; requires precise fixturing	Medium to high (laboratory or pilot stage)
Automated fibre placement (AFP)	High automation; accurate fibre placement; high repeatability; complex lay-ups; reduced human error; suitable for aerospace components; low core collapse and material wastage; mould-free	Very high equipment cost; slow deposition rates for thick parts; secondary bonding to cores often required; not cost-effective for large volumes	Low to medium (laboratory or pilot stage)
Additive manufacturing (AM)	High design freedom; integrated core/skin structures; mould-free; minimal waste; suitable for custom geometries	Low production rate; limited material choices; anisotropic properties; high cost per part; challenging surface finish	Very low (R&D, customized parts)
Thermoforming	Very short cycle times; low scrap rate; low part cost at high volumes; suitable for automation; good surface finish; scalable; compatible with large production lines	Limited thickness control; spring-back; restricted geometrical complexity; requires pre-consolidated laminates; less suitable for highly curved or complex shapes	Very high (mass production)

**Table 3:** Foaming routes and their corresponding properties.

Foaming Routes	Foaming Types	State Before Foaming	Polymer State While Foaming	Melt Strength of the Polymer	Cell Morphology	Typical Equipment
Batch foaming	Pressure-induced, Temperature-induced, Modified pressure-induced	Solid sheets, films, or beads	Polymer softened or partially melted; swelling occurs as gas forms	Medium to high	Uniform, mostly closed-cells	Autoclave or Compression moulding press
Semi-continuous or injection foaming	Mucell process, Core-back process	Melted polymer	Fully molten with blowing agent	Medium-low	Open-cells	Injection moulding machine
Continuous or extrusion foaming	Extrusion foaming, underwater granular	Melted polymer	Fully molten with dissolved blowing agent	Medium-low	Poor cell morphology (standard), or uniform open cells (underwater granulator)	Extruder

## Data Availability

No new data were created or analyzed in this study. Data sharing is not applicable to this article.
